# Antepartum Antibiotic Treatment Increases Offspring Susceptibility to Experimental Colitis: A Role of the Gut Microbiota

**DOI:** 10.1371/journal.pone.0142536

**Published:** 2015-11-25

**Authors:** Peris Mumbi Munyaka, N. Eissa, Charles Noah Bernstein, Ehsan Khafipour, Jean-Eric Ghia

**Affiliations:** 1 Department of Immunology, University of Manitoba, Winnipeg, Manitoba, Canada; 2 Department of Animal Science, University of Manitoba, Winnipeg, Manitoba, Canada; 3 Department of Internal Medicine, Section of Gastroenterology, University of Manitoba, Winnipeg, Manitoba, Canada; 4 Inflammatory Bowel Disease Clinical & Research Centre, University of Manitoba, Winnipeg, Manitoba, Canada; 5 Department of Medical Microbiology, University of Manitoba, Winnipeg, Manitoba, Canada; Charité, Campus Benjamin Franklin, GERMANY

## Abstract

**Background and aims:**

Postnatal maturation of the immune system is largely driven by exposure to microbes, and thus the nature of intestinal colonization may be associated with development of childhood diseases that may persist into adulthood. We investigated whether antepartum antibiotic (ATB) therapy can increase offspring susceptibility to experimental colitis through alteration of the gut microbiota.

**Methods:**

Pregnant C57Bl/6 mice were treated with cefazolin at 160 mg/kg body weight or with saline starting six days before due date. At 7 weeks, fecal samples were collected from male offspring after which they received 4% dextran sulfate sodium (DSS) in drinking water for 5 days. Disease activity index, histology, colonic IL-6, IL-1β and serum C-reactive protein (CRP) were determined. The V3-V4 region of colonic and fecal bacterial 16S rRNA was sequenced. Alpha-, beta-diversity and differences at the phylum and genus levels were determined, while functional pathways of classified bacteria were predicted.

**Results:**

ATB influenced fecal bacterial composition and hence bacterial functional pathways before induction of colitis. After induction of colitis, ATB increased onset of clinical disease, histologic score, and colonic IL-6. In addition, ATB decreased fecal microbial richness, changed fecal and colon microbial composition, which was accompanied by a modification of microbial functional pathways. Also, several taxa were associated with ATB at lower taxonomical levels.

**Conclusions:**

The results support the hypothesis that antepartum antibiotics modulate offspring intestinal bacterial colonization and increase susceptibility to develop colonic inflammation in a murine model of colitis, and may guide future interventions to restore physiologic intestinal colonization in offspring born by antibiotic-exposed mothers.

## Introduction

The use of antibiotics during pregnancy or around the time of delivery is a common practice in clinical settings in North America, more so due to the fear of newborn colonization with Group B streptococci (GBS) during passage through the birth canal or when the membranes rapture. The GBS is the leading cause of life-threatening neonatal bacterial infections in developed countries [1]. Efforts to prevent GBS infections in newborns [2] and to reduce the incidence of postpartum maternal infection after caesarean section [3] have led to the use of pre-delivery antibiotics in a large number of women in labor, resulting in exposure of the unborn fetus to the antibiotics. Although pre-partum antibiotics are generally recommended for premature rupture of membranes or when vaginal colonization by group B streptococci is detected, antibiotics are also frequently used in other clinical situations in which a clear benefit has not been demonstrated [4], and this has raised some concerns [5]. Also, even though antibiotics are designed to target bacterial pathogens, they often indiscreetly halt commensal human microbiota, allowing pathogens and opportunistic members of the bacterial community to propagate [6]. Perinatal antibiotics may also influence the initial microbial colonization of the newborn intestine, which is essential for the normal host development,[7] since the early neonatal period represents the most important opportunity for microbiota-induced host-homeostasis [8]. As such, intrapartum antibiotics have been associated with infant gut microbiota dysbiosis [9].

Antepartum antibiotics may affect the bacterial composition of the mother’s birth canal and or skin, which will be transmitted to the babies during and following delivery [10–12]. In this context, although antepartum antibiotics are short-term, they are given at a critical time when newborn acquisition of gut bacteria, which is also known to influence the initial immune system development, is just beginning. Thus, antepartum antibiotic exposure may have far-reaching implications on neonatal immune system maturation [13, 14]. The use of broad-spectrum antibiotics in the perinatal period has been shown to alter the expression of genes involved in gastrointestinal (GI) tract development, with major consequences on the architecture and functionality of the intestinal barrier [15]. Moreover, antibiotics given to pregnant mothers in the days before delivery have been shown to significantly alter the composition of the preterm newborn microbiota, reducing intestinal microbial diversity on the first stool samples [16]. It is therefore apparent that changes in the composition of the newborn indigenous microbiota may have the potential to influence childhood development and also their risk of disease [17].

The rapid increase in illnesses that have their onset in childhood (including asthma, allergies, type 1 diabetes, obesity and autism) suggests that an environmental cause could be present [13, 18, 19], and the loss of key constituents of the indigenous microbiota after maternal antibiotic exposure could be a contributing factor. For example, the use of antibiotics in the perinatal period have been associated with delayed colonization of neonates gut by several bacteria especially *Bifidobacteria* and *Lactobacillus* species [15, 20], and by decreased numbers of *Bifidobacteria* spp. and *Bacteroides* spp.[21]. This may have long-term impacts since these species are considered to have beneficial properties and therefore, their absence may predispose to infections. In this regard, increased incidence of atopic diseases, irritable bowel syndrome [22], and inflammatory bowel disease (IBD) have all been reported in antibiotic-exposed children [23–29].

Despite the high association between the impacts of perinatal or neonatal antibiotic use on the microbial colonization and future risk for asthma, other allergic reactions and disease conditions, the effects of antepartum antibiotic use on the process of intestinal microbiota development and future susceptibility to ulcerative colitis remain elusive or poorly understood. We assessed the susceptibility to colitis and compositional and functional alterations of fecal and colon mucosa-associated microbiota (MAM) in mice that were exposed to antepartum antibiotics and treated with dextran sulfate sodium (DSS) to induce acute colitis later in life.

## Materials and Methods

### Animals

Four pregnant C57Bl/6 mice were obtained from University of Manitoba breeding facility and maintained in the animal care facility at the Faculty of Health Sciences, University of Manitoba. These mice were treated with cefazolin (Midwest Veterinary Purchasing Cooperative Ltd, Winnipeg, MB, Canada) at 160 mg/kg/d (ATB group; 2 mice) or with saline (Control group; 2 mice) starting six days before due date. After delivery, the pups were left with their mothers until they were weaned on d 22. Upon weaning, the mice were housed in cages without mixing mice from different mothers and received a standard chow diet. The experimental protocol was approved by the University of Manitoba Animal Ethics Committee (10–073) and animals were cared for in accordance with the guidelines of the Canadian Council on Animal Care [30].

### Induction of DSS colitis

**Fig 1:**
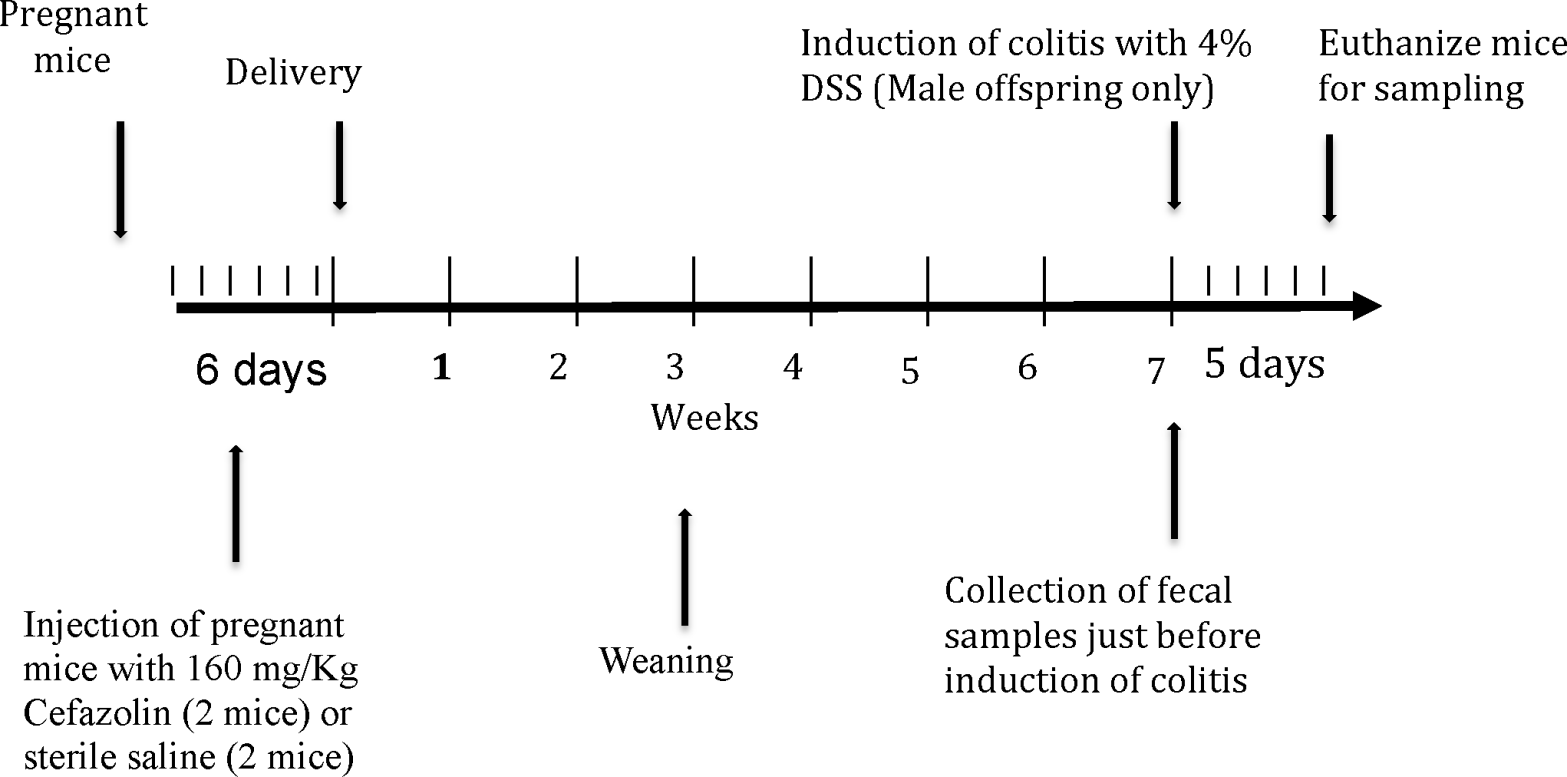
Experimental design and timelines for different experimental activities.

At 7 weeks of age, fecal samples were collected from male offspring from the two groups (ATB, n = 10 and Control, n = 10) after which they received DSS in their drinking water for 5 days (ATB-DSS, Control-DSS). The DSS (molecular weight; MW 40 kDa: MP Biomedicals, Soho, OH, USA) was added to the drinking water at a final concentration of 4% (wt/vol). Ten mice were included in each ATB-DSS and Control-DSS groups with five mice from each mother. **Fig 1**shows the experimental design and timelines for different activities.

### Evaluation of inflammation

During the period of colitis, the weights of the mice were recorded daily, and were expressed as a percentage of body weight prior to induction of colitis. Disease activity index comprised of the percentage of body weight lost score in combination with stool consistency, and blood in feces scores. The scoring system was defined as follows: Weight: 0, no loss; 1, 5–10%; 2, 10–15%; 3, 15–20%; and 4, >20%; stool: 0, normal; 2, loose stool; and 4, diarrhoea; and bleeding: 0, no blood; 2, presence of blood; and 4, gross blood, and the scores have historically correlated well with the pathological findings in DSS-induced model of colitis.[31]. The DAI scoring was performed from day 0 to day 5 over the period of DSS treatment. Presence of blood in the stool was assessed using the Hemoccult II test (Beckman coulter, Oakville, ON, Canada).

The colon was opened longitudinally and macroscopic damage was evaluated on the full section of the colon. The macroscopic scoring was performed immediately after the mice were sacrificed using previously established scoring system [31, 32], and the categories evaluated for macroscopic scores included, rectal bleeding, rectal prolapse, diarrhea and colonic bleeding.

For histology analysis and scoring, formalin (10%; Sigma, Mississauga, ON, Canada)-fixed colonic segments collected during sacrifice were paraffin (Sigma, Mississauga, ON, Canada)-embedded and 3-mm sections were stained using hematoxylin-eosin (H&E) (Sigma, Mississauga, ON, Canada). Colonic damage was assessed based on a published scoring system that considered loss of architectural, degree of inflammatory cell infiltrate, goblet cell depletion, and crypt abscess [31, 32].

For the analysis of serum C-reactive proteins, blood was collected by intracardiac puncture under isoflurane (Abbot, Mississaugua, ON, Canada) anaesthesia. Also, in order to assess colonic inflammatory cytokines, colonic samples were homogenized in 700 μl Tris-HCl buffer containing protease inhibitors (Sigma, Mississauga, ON, Canada), centrifuged at 13000 × *g* for 20 min at 4°C and the supernatant was frozen at—80°C until assay. Serum C-reactive protein (CRP) and colonic cytokine levels (IL-6, IL-1β) were determined using an ELISA commercial kit (R&D Systems, Minneapolis, MN, USA).

### DNA extraction and quality control

DNA was extracted from fecal and colon samples using ZR DNA extraction kits and quality was checked by PCR as described by Khafipour et al. [33]. Amplicons were verified by agarose gel electrophoresis. DNA extraction details are shown in S1 **Appendix**.

### Library construction and Illumina sequencing

The V3-V4 region of the 16S rRNA gene was targeted for PCR amplification using modified F338 primer (5´-AATGATACGGCGACCACCGAGATCTACACTATGGTAATTGTACTCCTACGGGAGGCAG-3´) for forward primer and modified bar coded R806 [34] as described previously [35]. S2 **Appendix** shows full library construction details, and the sequencing data are uploaded into the Sequence Read Archive (SRA) of NCBI (http://www.ncbi.nlm.nih.gov/sra) and can be accessed through accession number SRR2728570.

### Bioinformatic analyses

The FLASH assembler [36] was used to merge overlapping paired-end Illumina fastq files. The output fastq file was then analyzed by downstream computational pipelines of the open source software package QIIME (Quantitative Insights Into Microbial Ecology) [37]. Chimeric reads were filtered using UCHIME [38] and sequences were assigned to Operational Taxonomic Units (OTU) using the QIIME implementation of UCLUST [39] at 97% pairwise identity threshold. Taxonomies were assigned to the representative sequence of each OTU using RDP classifier and aligned with the Greengenes Core reference database [40] using PyNAST algorithms [41]. The phylogenetic tree was built with FastTree 2.1.3. [42] for further comparisons between microbial communities.

### Alpha-, beta-diversity analyses, partial least square discriminant analysis and metagenome prediction

Alpha-diversity was calculated using Chao 1 [43]. Beta-diversity was measured by calculating the unweighted and weighted UniFrac distances using QIIME [44] and the *P* values were calculated using PERMANOVA analyses of Bray-Curtis distances [45].

Partial least square discriminant analysis (PLS-DA; SIMCA P+ 14, Umetrics, Umea, Sweden) was performed on genus data to identify the effects of antepartum antibiotic treatment on the offspring as described previously [46]. Data was presented in loading scatter plots and the PLS-DA regression coefficients were used to identify taxa that were positively or negatively correlated with each treatment group. More details are shown in S3 **Appendix**.

Prediction of functional metagenome was done using the open source software PICRUSt [47] and STAMP [48] as described in S4 **Appendix**.

### Statistical analysis

Alpha-diversity and phylum percentage data were used to assess the effect of treatment using MIXED procedure of SAS (SAS 9.3). Disease activity index, weight loss, rectal bleeding and stool consistency were analyzed by applying two-way ANOVA followed by Sidak multiple comparison post hoc. Student *t* test was used to compare the macroscopic scores, histological scores and inflammatory markers between antibiotic and control groups using Graphpad Prism 5.0c (Graphpad Prism, La jolla, CA, USA). The significance level was adjusted at level 0.05.

## Results

### Fecal microbiota alterations before DSS treatment

As described below our results showed that antepartum use of antibiotics modified the ecology of offspring’s indigenous microbiota, and the effects persisted up to and possibly beyond seven weeks of age.

#### Alpha-diversity

**Fig 2:**
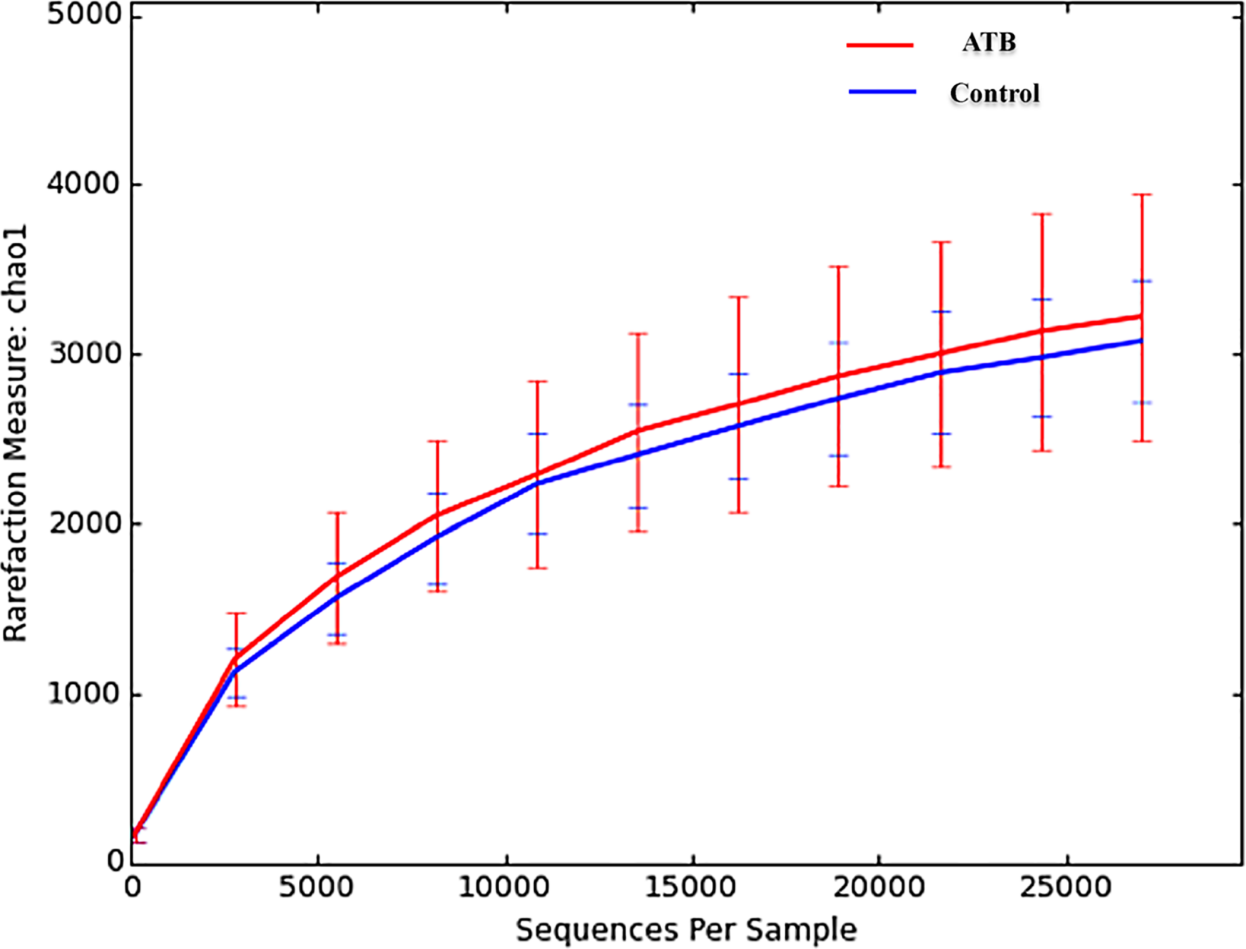
Alpha-diversity analysis on Chao 1, a measure of species richness based on operational taxonomic unit (OTU) for fecal samples collected before induction of colitis with DSS. No significance difference between the Control and the ATB group was observed. 10 mice per group.

As shown in **Fig 2**, there was no difference between ATB and control groups.

#### Beta-diversity

**Fig 3:**
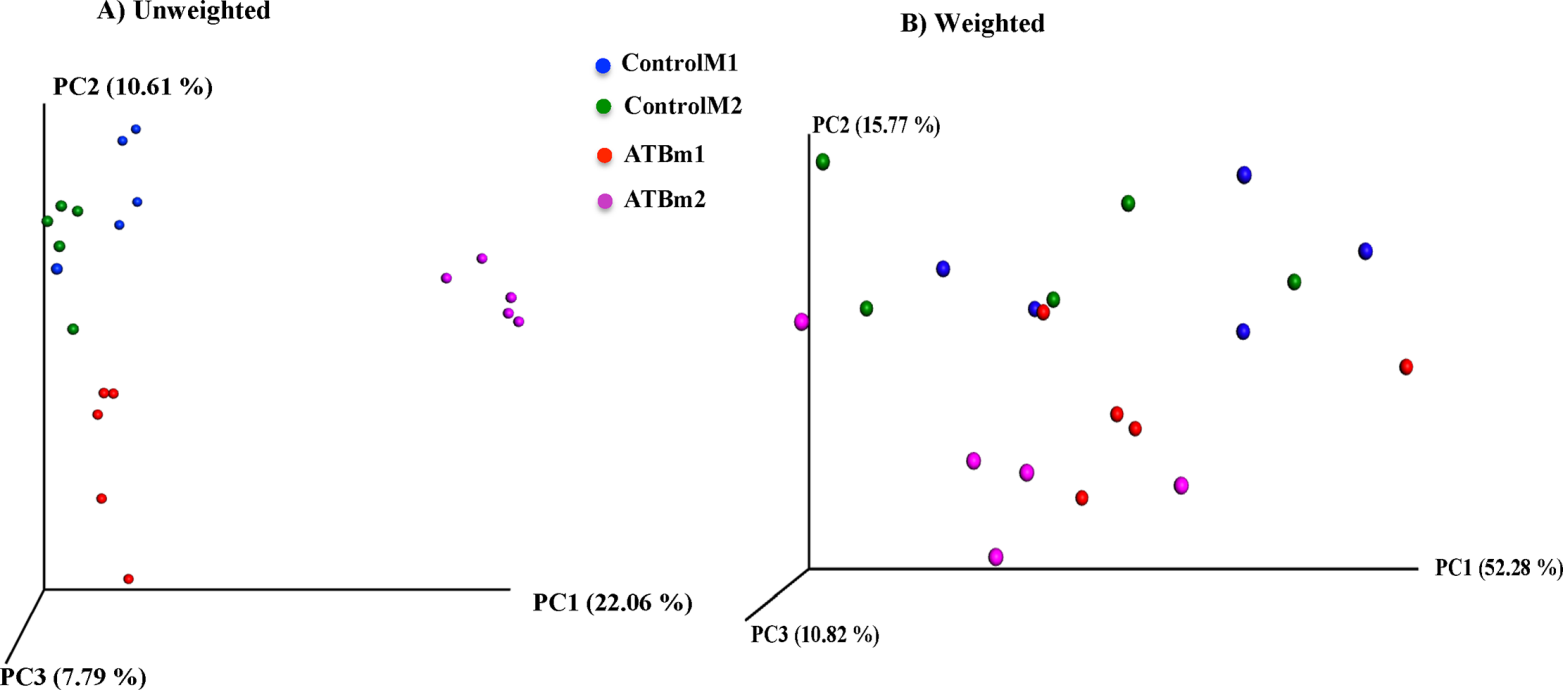
Principle coordinate analysis (PCoA) of (A) Unweighted (*P = 0*.*0003*) and (B) Weighted (*P* = 0.06) UniFrac distances, an OTU based unsupervised measure of beta-diversity in fecal samples collected before induction of colitis. Fecal samples clustered according to the treatment. The clustering was further influenced by the mother especially in the unweighted analysis (*P* = 0.001). The *P* values were determined using PERMANOVA. 10 mice per group. ControlM1 and ControlM2 shows mice in the control group but from two different mothers; ATBm1 and ATBm2 shows mice in the ATB (antibiotic) group but from two different mothers.

**Fig 3**presents the three-dimensional PCoA of unweighted and weighted UniFrac distances. Fecal samples were distinctly clustered according to their treatment group when plotted and analyzed using unweighted UniFrac (*P = 0*.*0003*). The clustering was not as distinct (*P = 0*.*06*) when PCOA and PERMANOVA analyses were performed on weighted UniFrac distances. The mother also influenced the clustering pattern of fecal samples and offspring from each mother clustered closer together in both ATB and Control groups. (*P* = 0.001).

#### Microbiota composition

**Fig 4:**
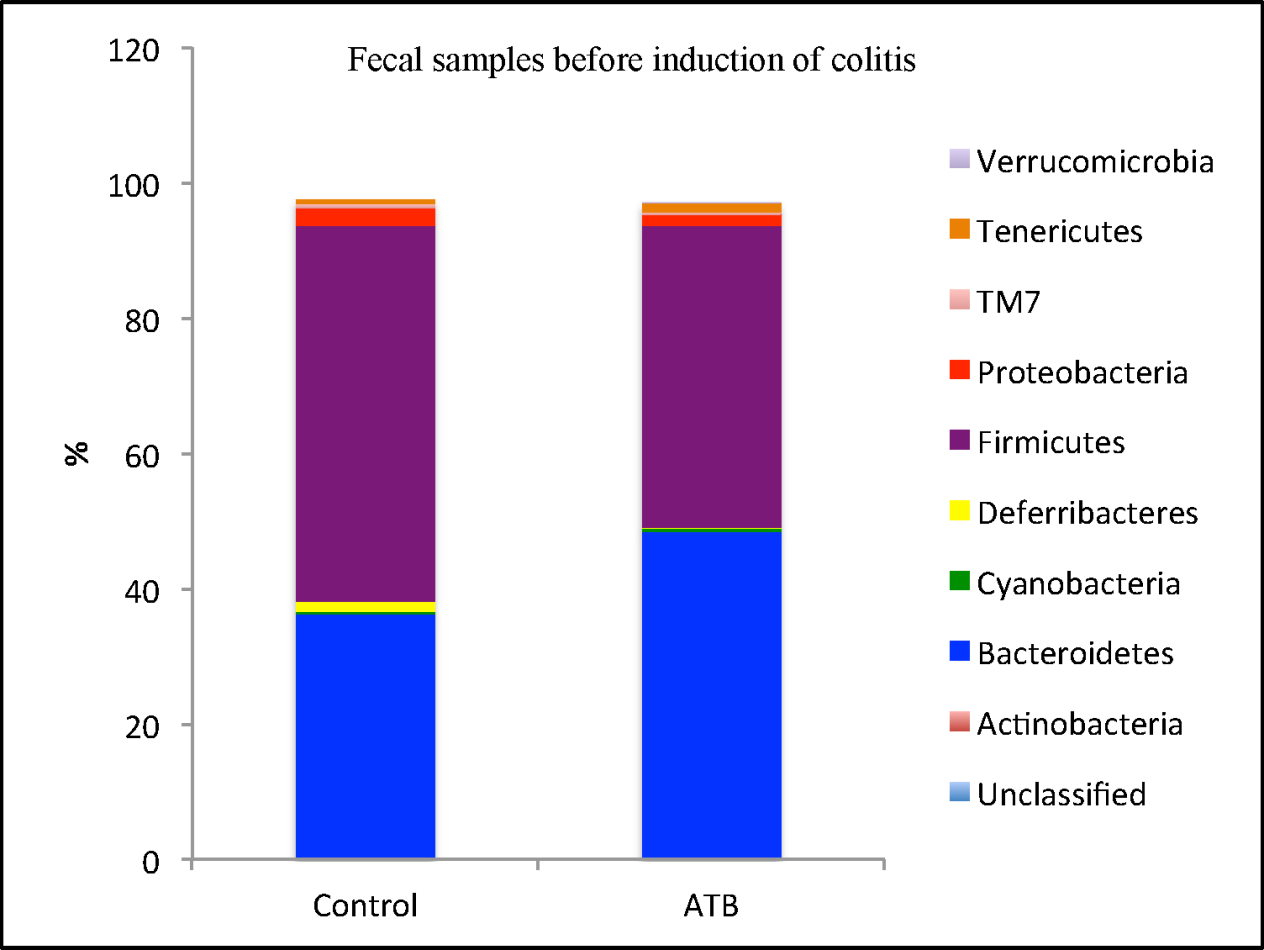
Relative abundances of bacterial phyla in fecal samples collected before induction of colitis. Antepartum antibiotic exposure significantly decreased Firmicutes and Proteobacteria but increased Bacteroidetes population. 10 mice per group.

A total of 9 phyla were identified, of which 3 were abundant (≥ 1% of population), including Firmicutes, Bacteroidetes and Proteobacteria. The low-abundance phyla (< 1% of population) included Actinobacteria, Cyanobacteria, Deferribacteres, Tenericutes, Verrucomicrobia and TM7. Among the abundant phyla, Firmicutes and Proteobacteria populations were lower (*P* = 0.01, and 0.04, respectively), while Bacteroidetes population was higher (*P =* 0.007) in the antibiotic group compared to the control (**Fig 4**).

**Fig 5:**
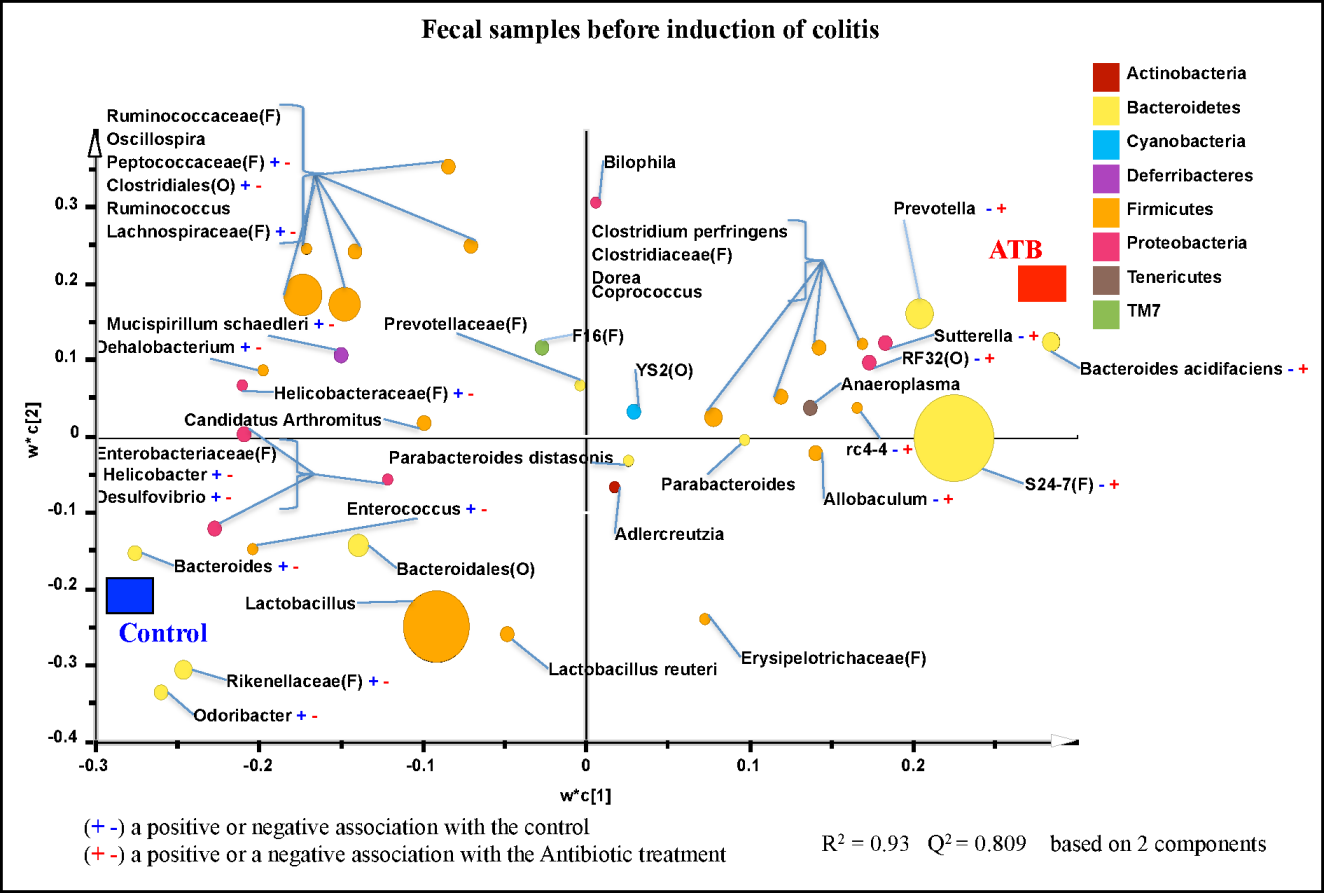
Partial least square discriminant analysis (PLS-DA) of bacterial communities comparing taxa that were associated with the Control or ATB treatments in the fecal samples collected before induction of colitis. All taxa are coloured based on the phyla to which they belong and sized based on their relative abundance. Some sequences could only be affiliated to phylum (p.), class (c.), order (o.), or family (f.) levels. Specific taxa were positively or negatively associated with each treatment group, which may be an indicator of shifts in the physiological or metabolic processes that may be influenced by the taxa. 10 mice per group.

Classification of the OTUs at the lower taxonomical levels resulted in identification of 102 taxa. Some taxa were only classified at the Phylum (p.), Class (c.), Order (o.), Family (f.), or Genus (g.) levels. Of the 102 taxa, 40 had abundances greater or equal to 0.01% of population, while 62 were below 0.01% of population. Bacterial taxa with relative abundance of ≥ 0.01% of population were analyzed using PLS-DA to identify bacteria that were most characteristic of the control or antibiotic groups. As shown in **Fig 5**, g. *Allobaculum*, *Bacteroides acidifaciens*, *Suterella*, *Prevotella*, *rc4-4*;, and unclassified members of f. S24-7; and o. RF32 were positively associated with antibiotic (ATB) group but negatively associated with control group. In addition, g. *Odoribacter*, *Bacteroides*, *Enterococcus*, *Desulfovibrio*, *Helicobacter*, *Dehalobacterium*, *Mucispirillum*; and unclassified members of f. Rikenellaceae, Helicobacteraceae, Lachnospiraceae, and Peptococcaceae were positively correlated with the control group but negatively associated with ATB treatment. **S1 Table** shows a summary of mean abundances of all the taxa.

#### Functional metagenome of fecal microbiome

**Fig 6:**
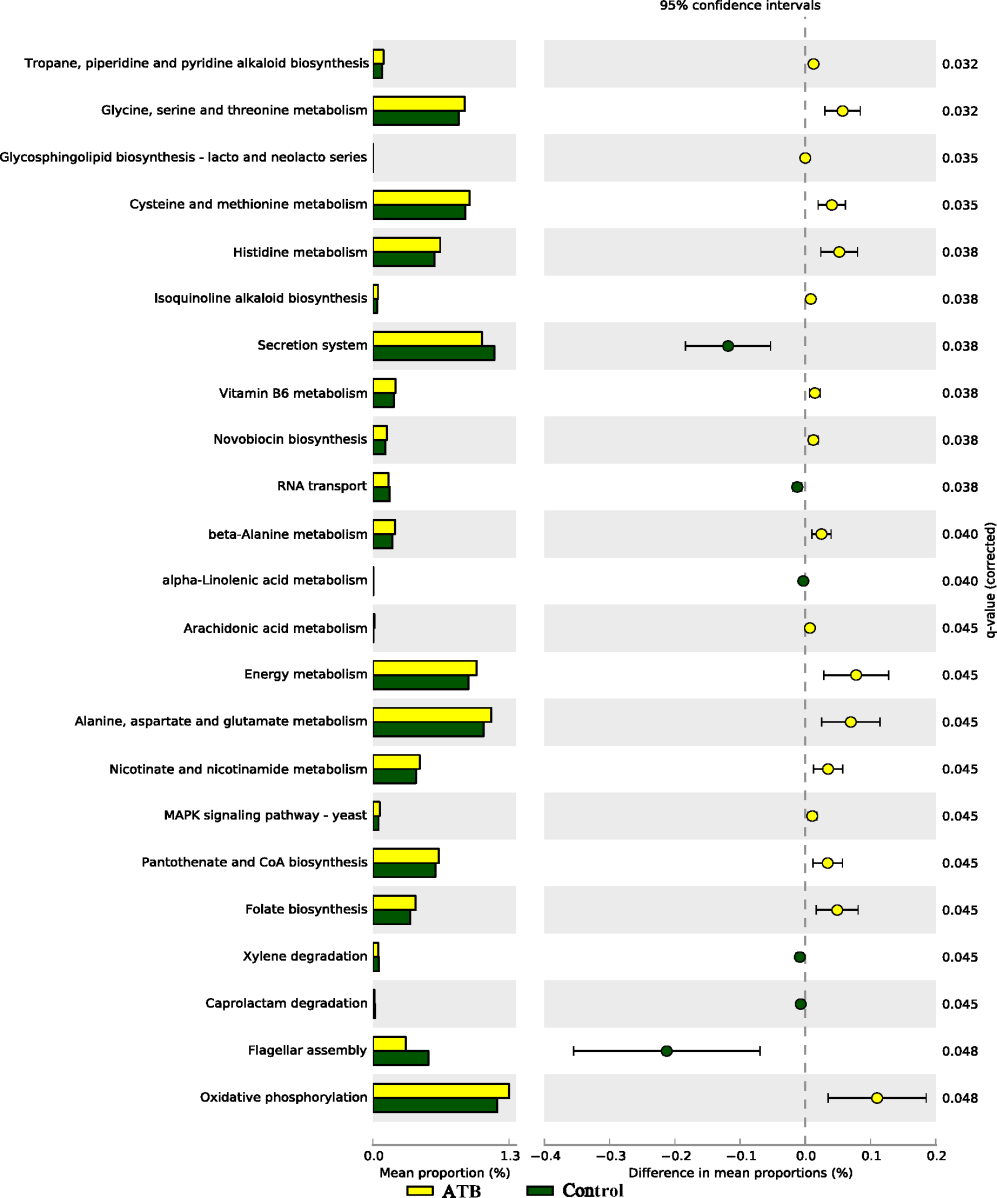
Subsystems and pathways enriched or decreased within fecal microbiome from Control and ATB groups before induction of colitis. Corrected *P* values were calculated using the Storey false discover rate (FDR) correction. Subsystems or pathways overrepresented in the ATB (Control) fecal samples have a positive (negative) difference between mean proportions and are indicated by white (black) color. 10 mice per group.

As shown in **Fig 6**, several pathways including oxidative phosphorylation, folate biosynthesis, pantothenate and CoA biosynthesis, energy metabolism, alanine, aspartate and glutamate metabolism, glycine, serine and threonine metabolism and histidine metabolism were highly enriched in the fecal microbiome of the ATB group compared to Control. In contrast, flagellar assembly and secretion system were highly enriched in the Control group compared to the ATB group.

### Impact of antepartum antibiotics on development of colitis

#### Disease activity index, macroscopic and histological scores

**Fig 7:**
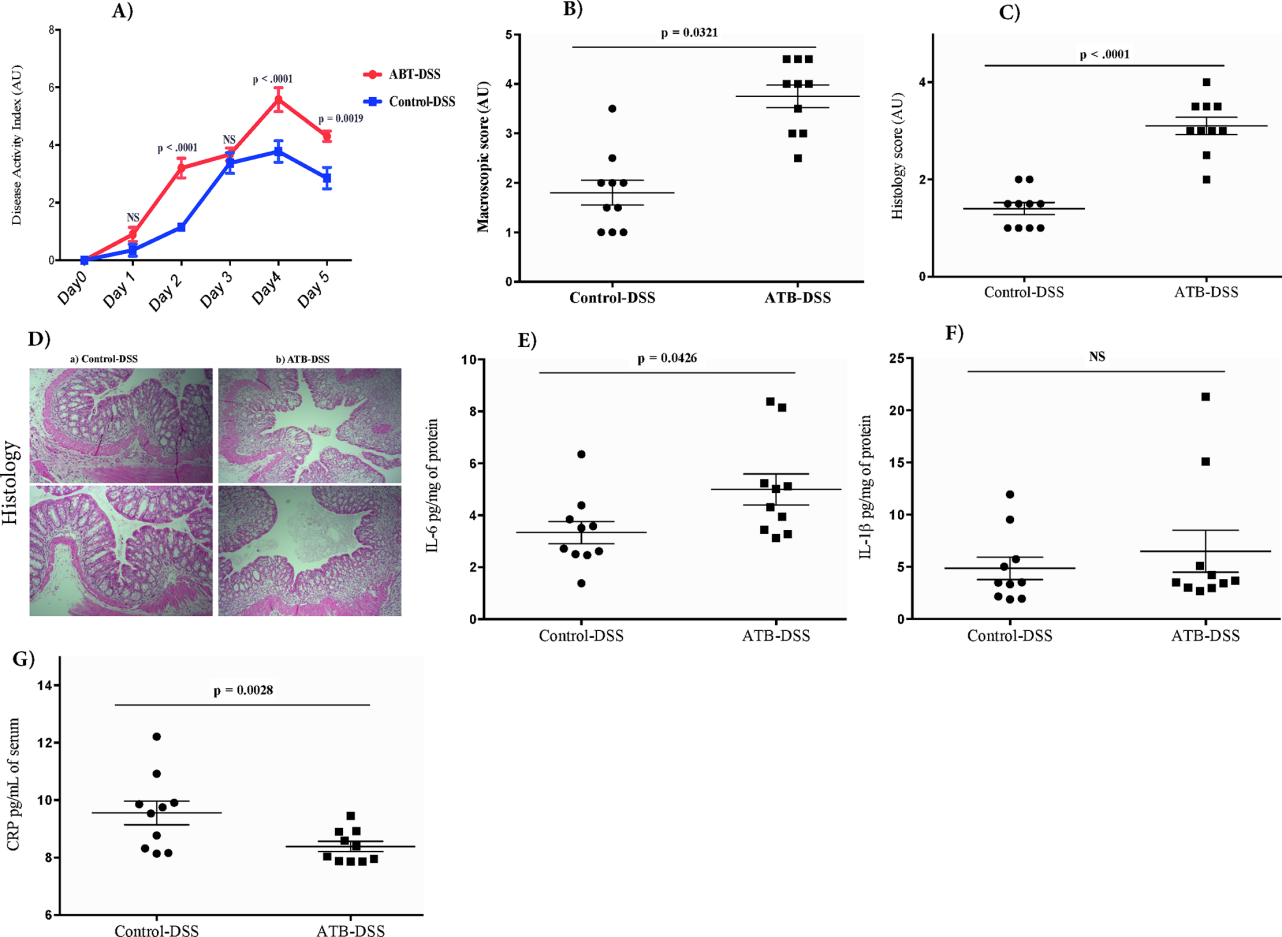
Impacts of dextran sulfate sodium (DSS) and antepartum antibiotics on colitis induction and disease severity. DSS caused a significant increase in the disease activity index **(A),** macroscopic score **(B)**, and histology score **(C)**. Antepartum antibiotic exposure increased histological score in DSS-induced colitis as assessed by appearance of colon **(D**) in mice without antibiotic exposure (Control-DSS) (**a**), and in mice exposed to antibiotics (ATB-DSS) (**b**). Antepartum antibiotic exposure increased IL-6 **(E)**, but did not effect IL-1β **(F**), and reduced C-reactive protein (CRP) **(G**). Values are shown as mean ± SEM. 10 mice per group.

Following DSS treatment, the onset of clinical disease, as assessed by disease activity index (stool consistency, weight loss and rectal bleeding) on d 2, 4, and 5 of the study increased in the ATB-DSS compared to the Control-DSS group (**Fig 7A**). **S1 Fig.** presents the stool consistency, weight loss and rectal bleeding. As shown in **Fig 7B**, DSS treatment increased colonic macroscopic damage score at d 5 in the ATB-DSS compared to the Control-DSS (*P* = 0.03). In addition, mucosal inflammation and infiltration was assessed through histological scoring as shown in **Fig 7C and 7D**, antepartum antibiotic exposure increased the severity of colitis associated with the loss of tissue architecture and increased immune cell infiltration.

#### IL-6, IL-1β, and C-reactive protein

Antepartum antibiotic exposure increased IL-6 levels (*P* = 0.04; **Fig 7E** in the colon of ATB-DSS mice compared to the Control-DSS, but did not affect IL-1β (**Fig 7F**), and reduced CRP levels (*P* = 0.002) (**Fig 7G**) in ATB-DSS compared to the Control-DSS.

### Colonic and fecal microbial alterations following DSS treatment

#### Colon and fecal alpha-diversity

**Fig 8:**
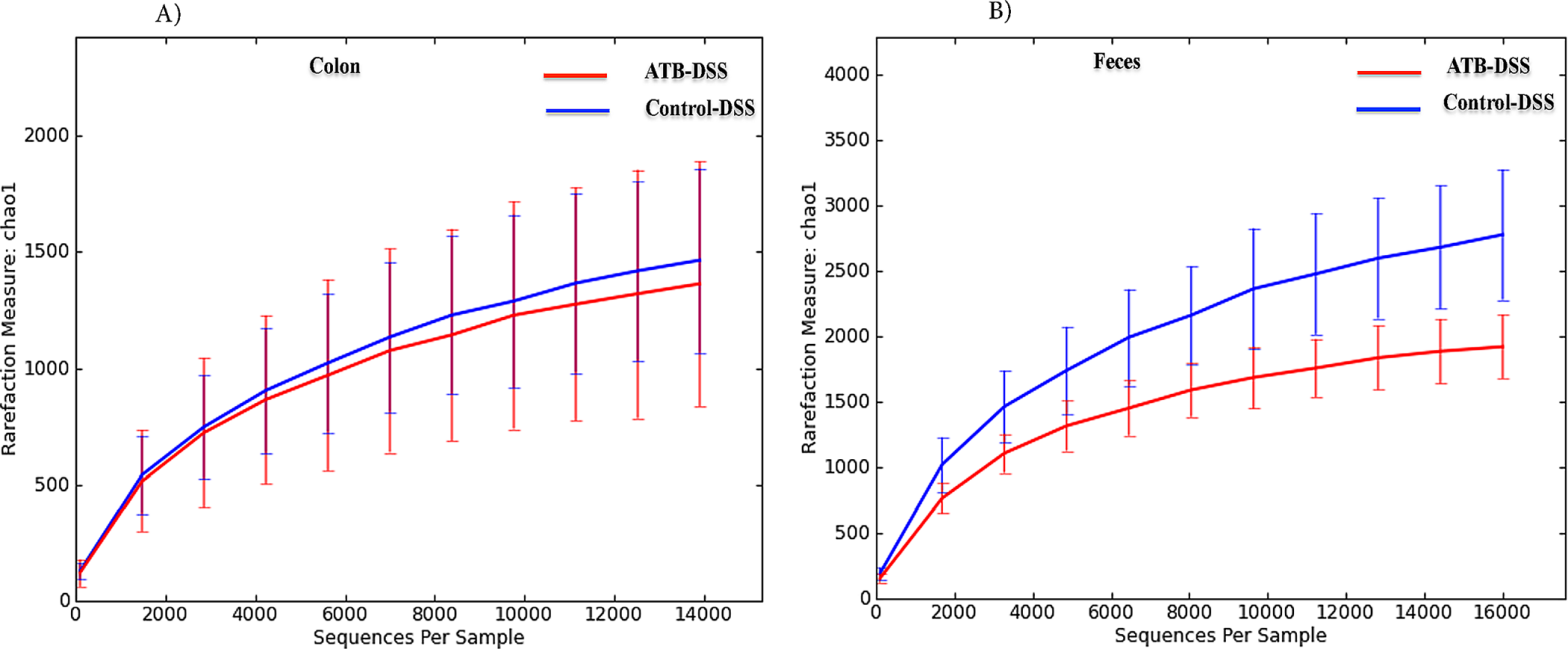
Rarefaction analysis on Chao 1, a measure of species richness based on operational taxonomic unit (OTU), following DSS treatment for the Control-DSS and ATB-DSS mice colon mucosa-associated (A) and fecal microbiota (B) Control-DSS and ATB-DSS had similar richness in the colon mucosa-associated microbiota; however, ATB-DSS had a lower fecal richness compared to the Control-DSS. 10 mice per group.

Antepartum antibiotic exposure did not influence colonic alpha-diversity (**Fig 8A**), but decreased fecal species richness (**Fig 8B**), 5 d after induction of colitis.

#### Colonic beta-diversity

**Fig 9:**
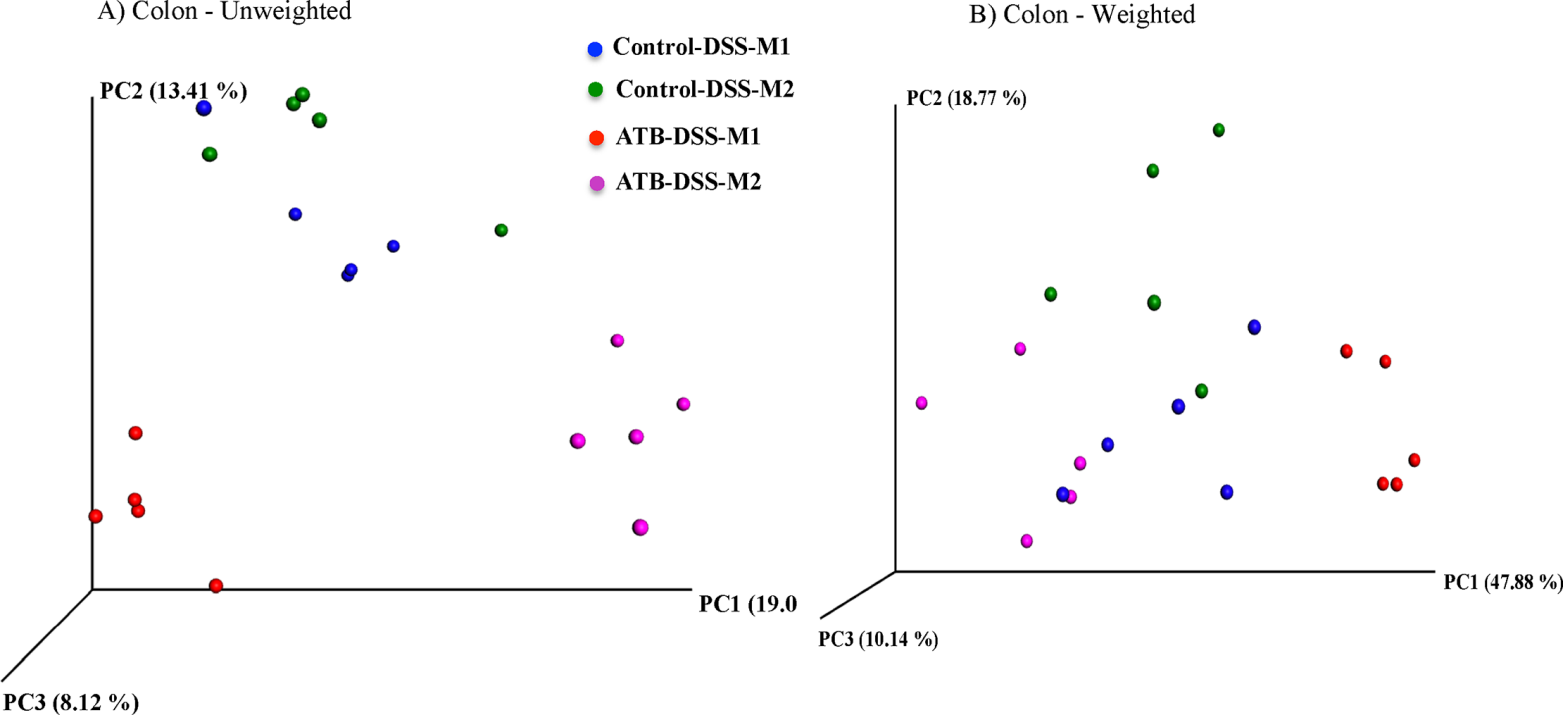
Principle coordinate analysis (PCoA) of (A) Unweighted (*P* = 0.0004) and (B) Weighted (*P* = 0.06) UniFrac distances in colon mucosa-associated microbiota (MAM) after induction of colitis. Colonic samples clustered according to the treatment. The clustering was further influenced by the mother (*P* < 0.001) especially in unweighted ATB-DSS (*P* = 0.009). The *P* values were determined using PERMANOVA. 10 mice per group. Control-DSS-M1 and Control-DSS-M2 shows mice in the control group but from two different mothers; ATB-DSS-M1 and ATB-DSS-M2 shows mice in the ATB (antibiotic) group but from two different mothers.

**Fig 9**shows a three-dimensional PCoA of unweighted and weighted UniFrac distances. Colonic samples clustered separately according to their treatment group when plotted and analyzed using unweighted UniFrac (*P* = 0.0004). The clustering was not as distinct (*P* = 0.06) on weighted UniFrac distances. The mother also influenced the clustering pattern of colon samples and offspring from each mother clustered closer together (*P* < 0.001), which was more evident in the ATB-DSS group compared to the Control-DSS group (*P* = 0.009).

#### Fecal beta-diversity

**Fig 10:**
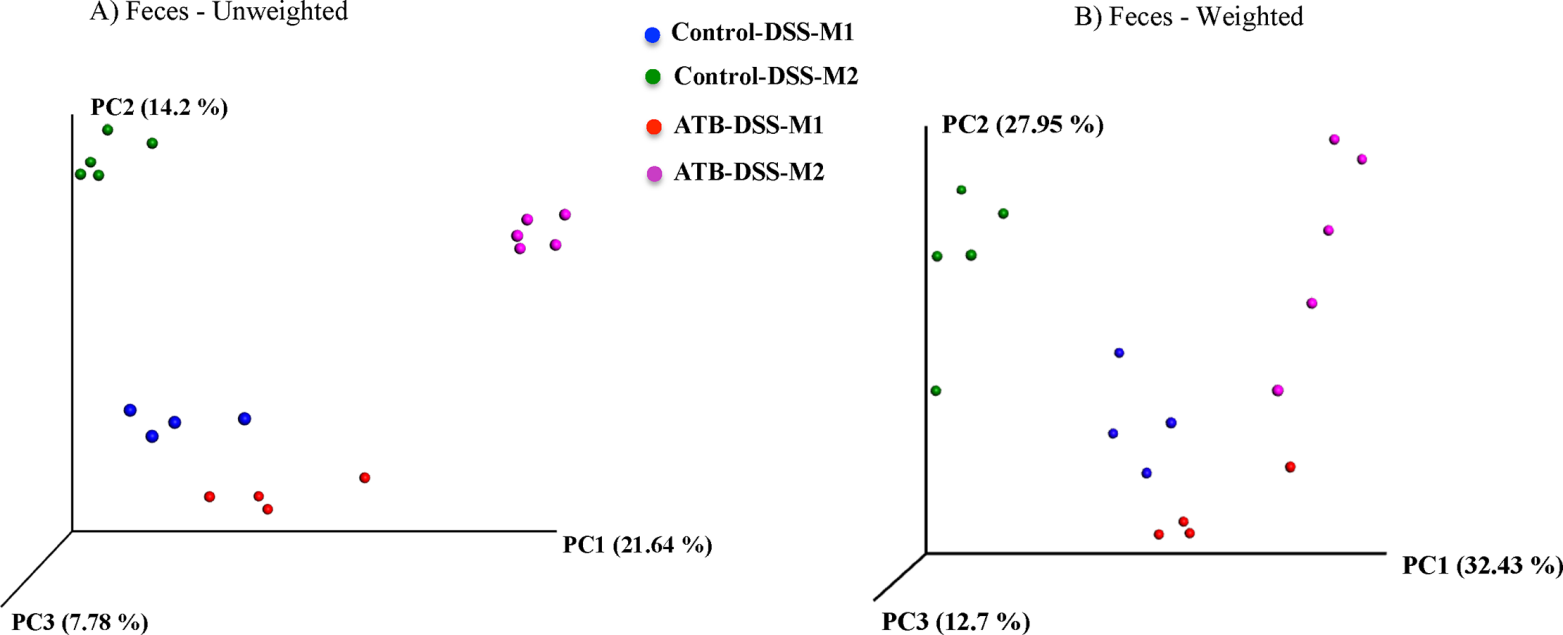
Principle coordinate analysis (PCoA) of (A) Unweighted (*P* = 0.0001) and (B) Weighted (*P* = 0.0001) UniFrac distances in fecal microbiota after induction of colitis. Fecal samples clustered according to the treatment and the mother (*P* = 0.001) further influenced the clustering. The *P* values were determined using PERMANOVA. 10 mice per group (two samples were excluded due to very low sequencing depth). Control-DSS-M1 and Control-DSS-M2 shows mice in the control group but from two different mothers; ATB-DSS-M1 and ATB-DSS-M2 shows mice in the ATB (antibiotic) group but from two different mothers.

As shown in **Fig 10**, fecal samples clustered distinctly according to treatment group for both unweighted and weighted UniFrac distance analysis (*P* = 0.0001). The mother also influenced the clustering as offspring samples from each mother clustered closer to each other (*P* = 0.001) in both the ATB-DSS and the Control-DSS groups. Two samples were omitted from the analysis due to very low sequencing depth.

#### Microbiota composition at phylum level in the colon and fecal samples

**Fig 11:**
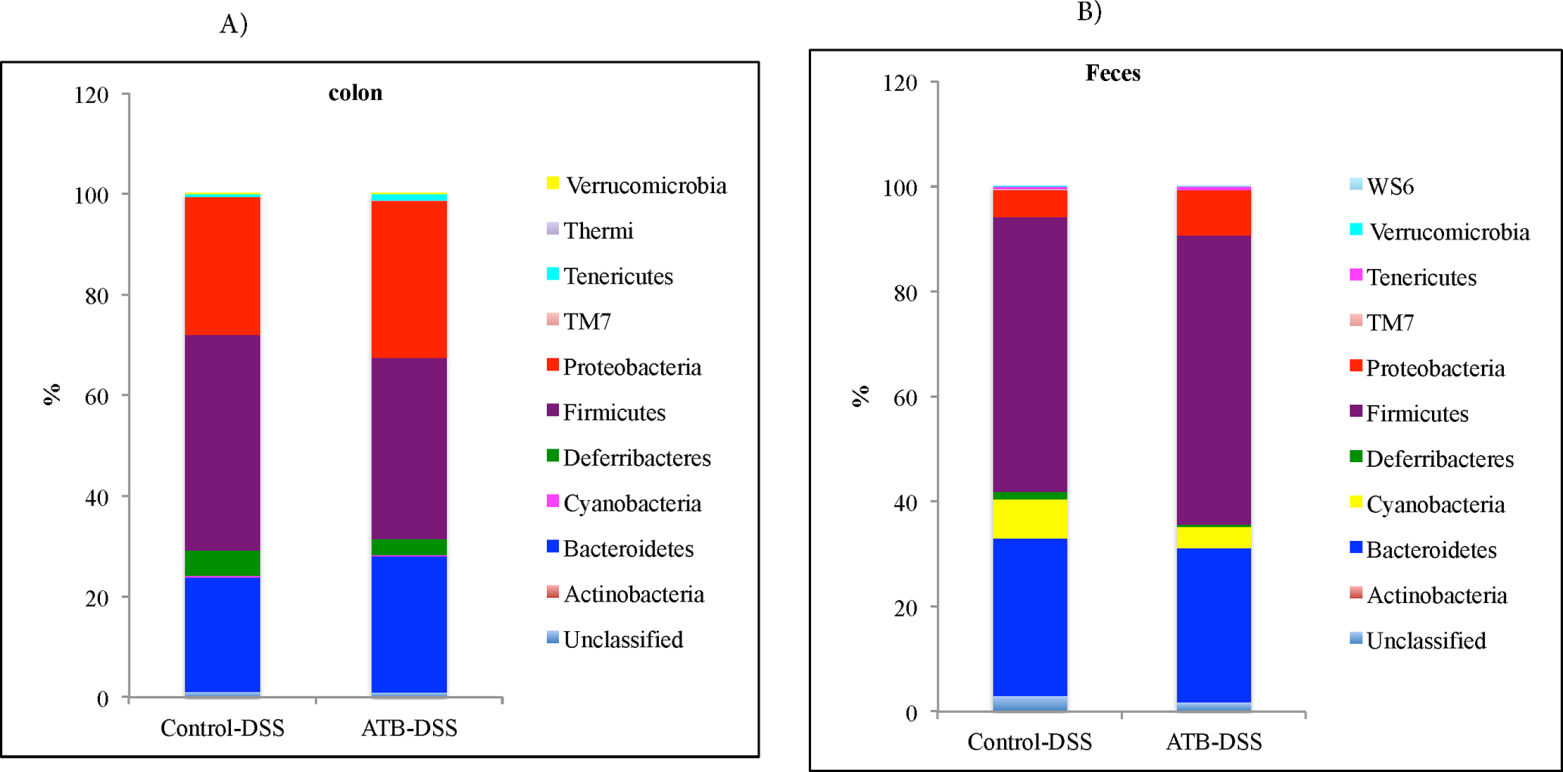
Relative abundances of bacterial phyla after induction of colitis in the colon mucosa (A) and in the feces (B). No significant difference was observed in the abundant phyla between the treatment groups. 10 mice per group.

A total of 10 phyla were identified in the colon mucosa samples, of which 4 were abundant (≥ 1% of population), including: Firmicutes, Bacteroidetes, Proteobacteria, and Deferribacteres. The low-abundance phyla (< 1% of population) included Actinobacteria, Cyanobacteria, Tenericutes, Thermi, Verrucomicrobia and TM7. No difference was observed between the antibiotic and the control group among the abundant phyla (**Fig 11A**).

In the fecal samples, a total of 10 phyla were identified, of which 4 were abundant (≥ 1% of population), including: Firmicutes, Bacteroidetes, Proteobacteria, and Cyanobacteria. The low-abundance phyla (< 1% of population), included Actinobacteria, Deferribacteres, Tenericutes, WS6, Verrucomicrobia and TM7. No significant difference was observed between the ATB-DSS and the Control-DSS group among the abundant phyla (**Fig 11B**).

#### Microbiota composition at lower taxonomic levels in the colonic samples

**Fig 12:**
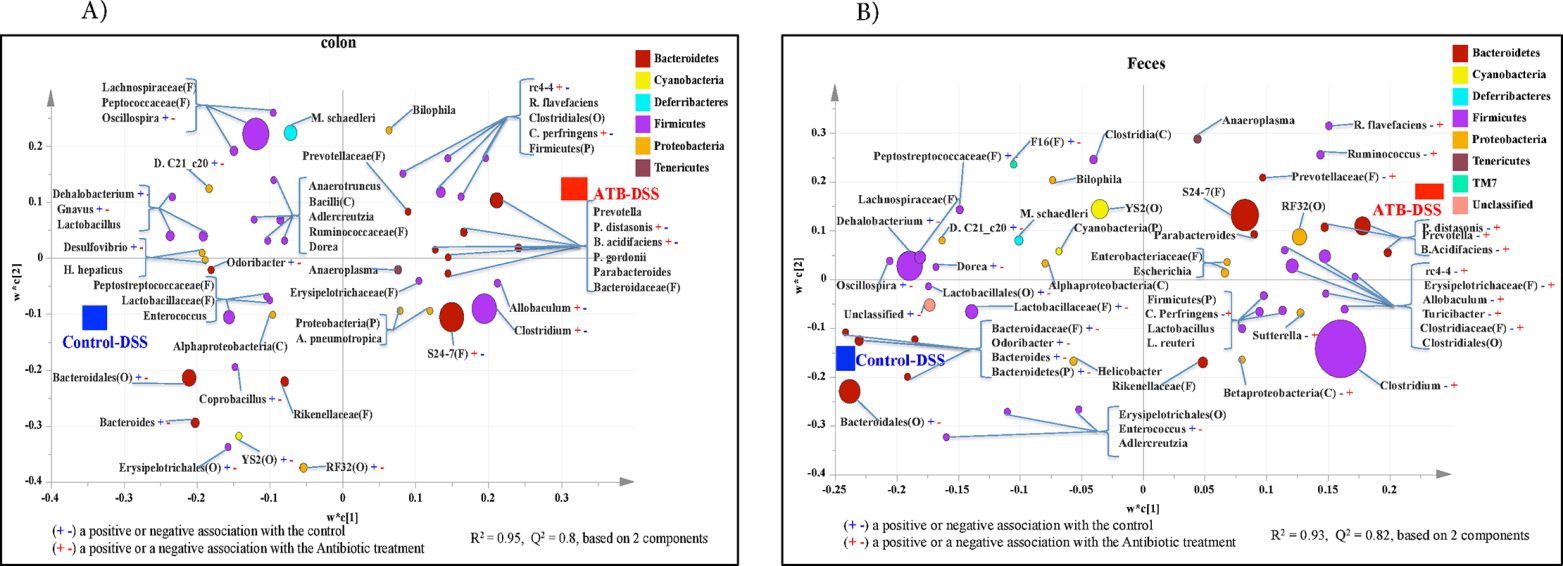
Partial least square discriminant analysis (PLS-DA) of bacterial communities comparing taxa that were associated with the Control-DSS or ATB-DSS treatments in the A) Colon mucosa, and B) Feces. All taxa are colored based on the phyla to which they belong and sized based on their relative abundance. Some sequences could only be affiliated to phylum (p.), class (c.), order (o.), or family (f.) levels. Specific taxa were positively or negatively associated with each treatment group, which may be an indicator of shifts in the physiological or metabolic processes that may be influenced by the taxa. 10 mice per group.

Classification of the OTUs at the lower taxonomical levels resulted in identification of 93 taxa. Of the 93 taxa, 60 had abundance grater than or equal to 0.01% of population, while 33 were below 0.01%. Bacterial taxa with relative abundance of ≥ 0.01% of population were analyzed using PLS-DA to identify bacteria that were most characteristic of the Control-DSS or ATB-DSS groups. As shown in **Fig 12A**, g. *Clostridium*, *Allobaculum*, *Bacteroides acidifaciens*, *Parabacteroides distasonis*, *Clostridium perfrigens*, *rc4-4*; and unclassified members of f. S24-7 were positively associated with the ATB-DSS group but negatively associated with the Control-DSS group. In addition, g. *Bacteroides*, *Coprobacillus*, *Odoribacter*, *Desulfovibrio*, *Gnavus*, *Dehalobacterium*, *Oscillospira*, *Desulfovibrio C21_c20*; and unclassified members of o. RF32, YS2, Erysipelotrichales, and Bacteroidales were positively associated with the Control-DSS group but negatively associated with the ATB-DSS group. **S2 Table** shows a summary of mean abundances of all the taxa.

#### Microbiota composition at lower taxonomic levels in the fecal samples

Classification of the OTUs at the lower taxonomical levels resulted in identification of 87 taxa. Of the 87 taxa, 55 had abundances greater than or equal to 0.01% of the population whilst 32 taxa were below 0.01% of the population. Bacterial taxa with relative abundance of ≥ 0.01% were analyzed using PLS-DA to identify bacteria that were most characteristic of the control or antibiotic groups. As shown in **Fig 12B**, g. *Clostridium*, *Betaproteobacteria*, *Sutterella*, *Clostridium perfrigens*, *Turicibacter*, *Allobaculum*, *rc4-4*, *B*. *acidifaciens*, *Prevotella*, *Parabacteroides distasonis*, *Ruminococcus*, *Ruminococcus flavefaciens;* unclassified members of f. Prevotellaceae, Erysipelotrichaceae, Clostridiaceae; and o. Clostridiales were positively associated with the ATB-DSS group but negatively associated with the Control-DSS group. Also, g. *Enterococcus*, *Bacteroides*, *Odoribacter*, *Dorea*, *Dehalobacterium*, *Desulfovibrio C21_c20*; unclassified members of f. Bacteroidaceae, Lactobacillaceae, Peptostreptococcaceae, F16; o. Lactobacillales; and p. Bacteroidetes were found to be positively correlated with the Control-DSS but negatively correlated with the ATB-DSS group. **S3 Table** shows a summary of mean abundances of all the taxa.

#### Functional metagenome of colonic and fecal microbiome

**Fig 13:**
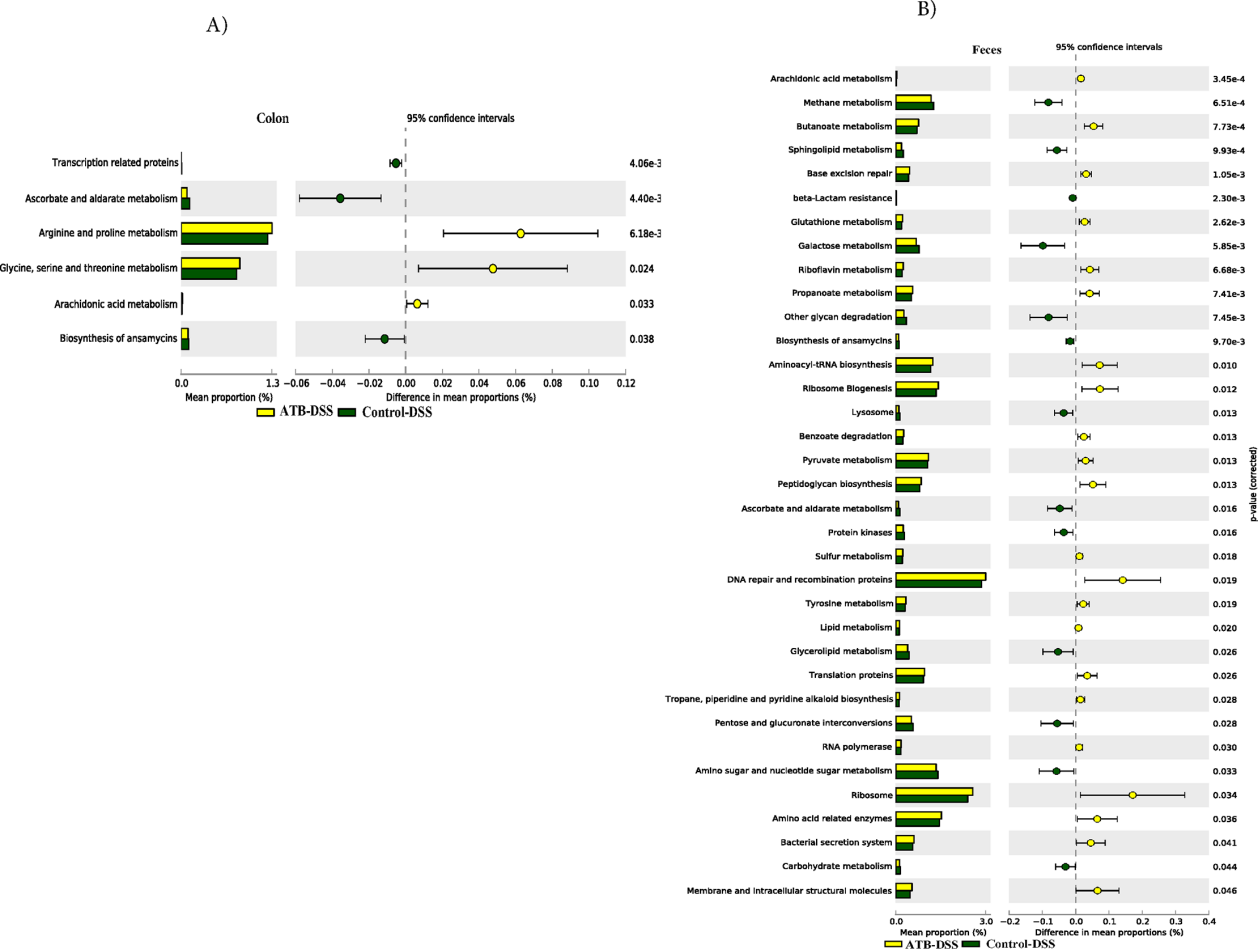
Subsystems and pathways enriched or decreased within (A) Colon muscosa-associate microbiome, and (B) Fecal microbiome after induction of colitis in Control-DSS and ATB-DSS. Subsystems or pathways overrepresented in the ATB-DSS (Control-DSS) fecal or colon mucosa samples have a positive (negative) difference between mean proportions and are indicated by white (black) color. 10 mice per group.

As shown in **Fig 13A and 13B**, several metabolic pathways including: arachidonic acid metabolism, butanoate, ribosome biogenesis metabolism, ribosome, peptidoglycan biosynthesis, carbohydrate digestion and absorption, arginine and proline metabolism, glycine, serine and threonine metabolism, were highly enriched in the mucosal microbiota of colon and in the feces in the ATB-DSS group.

## Discussion

The use of antibiotics may disrupt neonatal gut microbiota and have profound consequences for later health [20]. In this context, antibiotic-mediated disturbance of the intestinal microbiota in very early life has been shown to increase the risk of late-onset sepsis in a mouse model [49]. In addition, various illnesses with onset in childhood such as asthma, allergies, type 1 diabetes, obesity and autism have been hypothesized to be associated with maternal exposure to antibiotics resulting in perturbations of the indigenous microbiota [13, 18, 19]. Here, we assessed the effects of antepartum antibiotic (cefazolin) exposure of the mother on the gut microbiota composition of the offspring without or with exposure to experimental colitis later in life. Cefazolin belongs to the beta-lactam class and have a greater activity on gram-positive than gram-negative bacteria with a bactericidal effect inhibiting cell envelope synthesis [50, 51], and it was recently shown to be among the commonly administered intrapartum antibiotics in retrospective and prospective cohort studies conducted in Canada [9, 52].

Despite the smaller sample size in our study, we have demonstrated that at seven weeks of age, antepartum antibiotic treatment altered offspring fecal bacterial diversity, its composition at the phylum and lower taxonomical levels, and its predicted functional genome content. In addition, the characterization of the disease progress and severity demonstrated increased susceptibility to colitis in mice whose mothers were exposed to antepartum antibiotics compared to the mice whose mothers did not receive antibiotics. Similarly, the pattern of fecal microbiota was more profoundly altered following colitis induction in ATB-DSS mice compared to Control-DSS. It is important to note that all mice from the ATB and Control groups remained with their mothers and were exclusively suckling until the time of weaning (d 22). In addition, the mice from each mother were caged separately (i.e., mice from different mothers within the same group were not mixed at any point). Exclusive suckling, interactions with the mother, and the separate caging are all confounding factors, which have a direct influence on the nature of microbial colonization and might therefore complicate the interpretation of the data. Nevertheless, this may explain our observation on beta-diversity of fecal microbiota where both the antibiotic status and the mother seemed to influence sample clustering, suggesting that in addition to the effect of the antepartum antibiotics, mice from different mothers had distinct bacterial composition. In support of this, intrapartum antibiotics have been associated with infant gut microbiota dysbiosis, and breastfeeding was found to modify the effects [9]. With respect to colon MAM, the effect of mother on bacterial composition was not as apparent in the Control-DSS mice compared to the ATB-DSS mice. Although the differences between colon MAM and fecal microbiota composition are well established [53], the reasons why colon MAM in the Control-DSS was not influenced by the mother as opposed to that of fecal microbiota are not clear and remain to be explained.

Mice whose mothers received antepartum antibiotics had an increased disease activity index on d 2, 4 and 5 of DSS treatment, as well as increased macroscopic score on d 5 and an increased level of colonic IL-6 compared to the DSS mice with no antibiotics. The histological score also revealed destruction of the colonic wall characterized by a loss of crypts. However, IL-1 level was not modified whereas serum CRP, a marker of systemic inflammation decreased in the antibiotic group. These results suggest an increased activity of DSS because of antepartum antibiotic exposure, as assessed by disease severity and colonic damage, compared to the control. This is in agreement with other studies where the use of broad-spectrum antibiotics in the antepartum period was shown to alter expression of genes involved in gastrointestinal tract development, particularly the architecture and functionality of the intestinal barrier [15]. It is important to emphasize, however, that the course of systemic inflammation as measured by CRP showed different response as the ATB-DSS group had lower level of CRP compared to that of Control-DSS group, which is in contrast to histological and disease severity indices, suggesting a different mode of action. Nevertheless, although this phenomenon is not clear to us, intravenous administration of cefazolin was previously shown to lower CRP to normal levels in a patient with elevated levels of CRP [54].

Our data showed that fecal bacterial species richness did not differ between the Control and ATB mice before induction of colitis although microbiota composition was different; however, after 5 days of DSS administration, ATB-DSS mice had a lower fecal bacterial richness, suggesting that the antepartum antibiotic exposed mice were more susceptible to colitis compared to the control mice that were equally treated with DSS. Also, both colon MAM and fecal microbiota differed in ATB-DSS mice compared to Control-DSS. Direct exposure to antibiotics is known to affect intestinal colonization by suppressing commensal bacteria and causing the emergence of pathogens such as *Clostridium difficile* [55]. Research shows that antibiotic use in the immediate period after birth can severely alter gut microbiota in infants [21, 56], and evidence from long-term studies suggests that these perturbations could last for months, if not years [57, 58]. Indirect exposure is also relevant, because gut microbial diversity was reduced in infants born to mothers who received antibiotics during pregnancy or while breastfeeding [59], which is in agreement with our results. The finding that fecal bacterial species richness did not differ before induction of colitis but the antibiotic group had a lower species richness following administration of DSS, suggests a role of antepartum antibiotics in susceptibility to DSS-induced microbial dysbiosis.

Firmicutes, Bacteroidetes, Proteobacteria and Actinobacteria are usually the most abundant phyla in a healthy gut and in most cases Firmicutes and Bacteroidetes are depleted whereas Actinobacteria and Proteobacteria substantially become more abundant in IBD patients compared to healthy controls [60]. Although we did not observe differences between antibiotic and non-antibiotic groups at the phylum level 5 days after induction of colitis, we found that specific taxa were associated with each group at lower taxonomical levels. However, data on the indirect impact of antepartum antibiotic use on offspring gut microbiota colonization is not consistent. In this regard, previous studies did not observe effect of maternal antibiotics during pregnancy upon infant gut microbiota [21], whereas others reported an effect of maternal perinatal antibiotics use on fecal microbiota or first stool sample, such as reduced intestinal microbial diversity and shifts in abundance of specific bacteria in both full-term and pre-term infants [16, 59, 61]. In agreement with our observations, intrapartum antimicrobial prophylaxis was also found to have an equal or even higher effect on intestinal microbiota in infants during the first days of life compared to direct antibiotic administration, even when the mothers received only a single dose of ampicillin [61].

The analysis of microbiota profile in fecal samples before the induction of colitis provided an opportunity to address a relevant question regarding antepartum antibiotics. In this context, the results support the hypothesis that antepartum antibiotics indeed do perturb the initial establishment of gut microbiota in offspring and therefore, provide an opportunity to further establish whether these perturbations have any role to play in susceptibility to colitis. The fecal bacterial community composition was altered as a result of antepartum antibiotics and these alterations were even more pronounced after treatment with DSS. The observed microbial changes because of the antepartum use of cefazolin could be driven by the presence/absence and enrichment/depletion of specific taxa within certain phyla. In this regard, several genera including *Clostridium*, *Allobaculum*, and *rc4-4*, and species, such as *Bacteroides acidifaciens*, *Parabacteroides distasonis*, *Clostridium perfrigens* were positively associated with the antibiotic group in both colon MAM and fecal microbiota. There were several other taxa that were positively associated with the antibiotic group either only in the colon (unclassified members of f. S24-7), or in the feces (g. *Ruminococcus flavefaciens*, *Betaproteobacteria*, *Sutterella*, *Prevotella*, *Ruminococcus* and *Turicibacter;* unclassified members of o. Clostridiales; f. Clostridiaceae, Erysipelotrichaceae, and Prevotellaceae). Of particular interest, g. *Bacteroides acidifaciens*, *Allobaculum*, *rc4-4*, *Prevotella*, and *Sutterella*, and f. S24-7 were also enriched in the feces of the ATB group before induction of colitis indicating their strong association with antepartum exposure to cefazolin which persisted even after DSS treatment, and therefore, it is speculated that these taxa may play significant roles in the susceptibility to colitis. For example, *Sutterella* is associated with low levels of IgA in the gut and just recently it was shown to have the capacity to degrade secretory IgA[62] that protects the mucosa and regulates microbial attachment in the mucosa. As such, degradation of secretory IgA by *Sutterella* which was more enriched both in ATB and ATB-DSS mice compared to Control and Control-DSS mice, may imply a less protected mucosa in mice whose mothers were exposed to antepartum antibiotics, and thus, render the mucosa susceptible to colitis.

Persaud et al [63]. examined the effects of antibiotic exposure in the perinatal period on the gut microbiota of 184 infants enrolled in the Canadian Healthy Infant Longitudinal Development Study. The study showed that infants had an increased relative abundance of *Clostridium* [63], emphasizing on the association of *Clostridium* with prenatal antibiotic use. Most members of Clostridium, including *Clostridium perfrigens* and *Clostridium difficile*, are known to be pathogenic and or opportunistic bacteria that are able to take advantage of gut microbiota dysbiosis following antibiotic exposure, facilitating their proliferation, and ability to occupy ecological niches previously unavailable for them [64]. Also, perinatal antibiotics have been shown to increase the abundance of Enterobacteriaceae family in infants [61]. In addition, antibiotic administration during the first hours of life increased the levels of Enterobacteriaceae as analyzed during the first 2 months of life [56, 65]. Similarly, incomplete recovery of the gut microbiota after a 5 d antibiotic administration has been demonstrated in adults aged 22–43 years [57]. These results suggest a lasting effect of antibiotic administration on gut microbiota composition that is likely to influence disease risk, which is in agreement with our observations. Based on our results, we can speculate that the taxa that were associated with the antibiotic group may have specific roles in the susceptibility to colitis. However, it is important to note that different bacteria exhibit redundancy in their functions and some may appear or disappear from the community depending on the existing conditions. Also, different antibiotics may exhibit different effects depending on which members of the commensal bacteria are targeted by the antibiotics.

Metagenome prediction revealed functional shifts in the murine intestinal microbiome, with different metabolic pathways enriched in the colon mucosa-associated microbiome and the fecal microbiome in the ATB group compared to the Control, although the range of functions that were impacted were greater in fecal compared to the colon mucosa-associated microbiome. Microbial functions related to arachidonic acid metabolism may play important roles in inflammatory responses through production of prostaglandins [66], while butanoate metabolism could be associated with the integrity of the colonocytes. Other functional pathways including: ribosome biogenesis and metabolism, carbohydrate digestion and absorption, arginine and proline metabolism, glycine, serine and threonine metabolism may be important in nutrient availability and synthesis of proteins, which may directly or indirectly influence the host. Apart from glycine, serine and threonine metabolism that was common in the antibiotic group before and after DSS treatment, the fecal microbial functional and metabolic activities in the ATB group were majorly different before and after induction of colitis suggesting that DSS further modified the community functional potential although this may also be influenced by interactions between indirect effects of cefazolin and DSS.

In this study, we used the antibiotic cefazolin as a tool to indirectly induce shifts in the intestinal microbiota and model an altered colonization state later in life. We have demonstrated a microbiota-driven, specific increase in susceptibility to experimental murine colitis and have provided data that suggests these effects could be mediated by changes in microbial colonization through antepartum antibiotic exposure. Although the mechanisms involved in cefazolin-mediated susceptibility of murine colitis remain to be directly elucidated, the data presented here provide new insights and offer avenues for future work. This new knowledge has extended our understanding of impact of antepartum antibiotics with respect to microbial dysbiosis and colitis and may form a basis for designing intervention strategies targeting the gut microbiota. This data also suggests that perinatal antibiotics may predispose offspring to colitis and thus, the potential deleterious effects upon gut microbiota composition may need to be considered when deciding on maternal antibiotic use.

## Supporting Information

S1 Appendix DNA extraction. (DOCX)

S2 Appendix Library construction, Illumina sequencing. (DOCX)

S3 Appendix Partial least square discriminant analysis and depth for alpha-diversity analysis. (DOCX)

S4 Appendix Prediction of functional metagenome. (DOCX)

S1 Fig Impacts of dextran sulfate sodium (DSS) and antepartum antibiotics on colitis induction and disease severity. In the ATB group, DSS caused a significant increase in diarreah **(A),** Blood in the feces **(B)** especially on day 2, 4, and 5, but did not significantly influence weight loss **(C).**
(DOCX)

S1 Table Mean relative abundance of taxa in fecal samples collected just before induction of colitis. Some taxa were only classified at the Phylum (p.), Class (c.), Order (o.), Family (f.), or Genus (g.) levels. Mean values only, no statistical analysis
(DOCX)

S2 Table Mean relative abundance of taxa in colon samples collected after induction of colitis. Some taxa were only classified at the Phylum (p.), Class (c.), Order (o.), Family (f.), or Genus (g.) levels. Mean values only, no statistical analysis
(DOCX)

S3 Table Mean relative abundance of taxa in fecal samples collected after induction of colitis. Some taxa were only classified at the Phylum (p.), Class (c.), Order (o.), Family (f.), or Genus (g.) levels. Mean values only, no statistical analysis.
(DOCX)

## Abbreviations

CRP: C-reactive protein
CD: Crohn’s disease
DSS: dextran sulfate sodium
DAI: disease activity index
IBD: inflammatory bowel diseases
IL: interleukin
PLS-DA: partial least square discriminant analysis
OTU: operational taxonomic unit
TNF: tumor necrosis factor
UC: ulcerative colitis
