# Supplementary material for: Antepartum Antibiotic Treatment Increases Offspring Susceptibility to Experimental Colitis: A Role of the Gut Microbiota
Source: PLoS One. 2015 Nov 25;10(11):e0142536. doi: 10.1371/journal.pone.0142536 (PMC4659638; doi:10.1371/journal.pone.0142536)
Supplement: S1 Appendix — (DOCX) [file pone.0142536.s001.docx]

**Supporting information**

**S1 Text. DNA extraction**

Approximately 200 mg of each fecal sample were used for DNA extraction using ZR fecal DNA extraction kit (Zymo Research Corp., Orange, CA, USA). For colonic samples, the tissue was cut open and approximately 50 mg of mucosa scrapings were taken. DNA extraction was done using ZR Tissue and Insect DNA kit (Zymo Research Corp., Orange, CA, USA). Both DNA extraction kits have bead-beating step for the mechanical lysis of the microbial cells. DNA was quantified using a NanoDrop 2000 spectrophotometer (Thermo Scientific, Wilmington, DE, USA). DNA samples were normalized to 20 ng/µl, and quality checked by PCR amplification of the 16S rRNA gene using universal primers 27F (5'-GAAGAGTTTGATCATGGCTCAG-3') and 342R (5'-CTGCTGCCTCCCGTAG-3').

^*^ Mean values only, no statistical analysis
