# Supplementary material for: Antepartum Antibiotic Treatment Increases Offspring Susceptibility to Experimental Colitis: A Role of the Gut Microbiota
Source: PLoS One. 2015 Nov 25;10(11):e0142536. doi: 10.1371/journal.pone.0142536 (PMC4659638; doi:10.1371/journal.pone.0142536)
Supplement: S2 Appendix — (DOCX) [file pone.0142536.s002.docx]

**Supporting information**

**S2 Text. Library construction, Illumina sequencing**

The PCR reaction for each sample was performed in duplicate and contained 1.0 µl of pre-normalized DNA, 1.0 µl of each forward and reverse primers (10 µM), 12 µl HPLC grade water (Fisher Scientific, Ottawa, ON, Canada) and 10 µl 5 Prime Hot MasterMix® (5 Prime, Inc., Gaithersburg, MD, USA). Reactions consisted of an initial denaturing step at 94°C for 3 min followed by 35 amplification cycles at 94°C for 45 sec, 50°C for 60 sec and 72°C for 90 sec; this was finalized by an extension step at 72°C for 10 min in an Eppendorf Mastercycler® (Eppendorf, Hamburg, Germany). The PCR products were then purified using ZR-96 DNA Clean-up Kit™ (ZYMO Research, Irvine, CA, USA) to remove primers, dNTPs and reaction components. The V3-V4 library was then generated by pooling 200 ng of each sample, quantified by Picogreen dsDNA (Invitrogen, Burlington, NY, USA). This was followed by multiple dilution steps using pre-chilled hybridization buffer (HT1) (Illumina, San Diego, CA, USA) to bring the pooled amplicons to a final concentration of 5 pM, measured by Qubit® 2.0 Fluorometer (Life technologies, Burlington, ON, Canada). Finally, 15% of the PhiX control library was spiked into the amplicon pool to improve the unbalanced and biased base composition, a known characteristic of low diversity 16S rRNA libraries. Customized sequencing primers for read1 (V4) (5'- TATGGTAATTGTGTGCCAGCMGCCGCGGTAA -3'), read 1 (V3-V4) 5'- TATGGTAATTGTACTCCTACGGGAGGCAG -3' read2 (5'- AGTCAGTCAGCCGGACTACHVGGGTWTCTAAT -3') and index read (5'- ATTAGAWACCCBDGTAGTCCGGCTGACTGACT -3') were synthesized and purified by polyacrylamide gel electrophoresis (Integrated DNA Technologies, Coralville, IA, USA) and added to the MiSeq Reagent Kit V3 (600-cycle) (Illumina, San Diego, CA, USA). The 150 and 300 paired-end sequencing reactions were performed on a MiSeq platform (Illumina, San Diego, CA, USA) at the Gut Microbiome and Large Animal Biosecurity Laboratories, Department of Animal Science, University of Manitoba, Winnipeg, MB, Canada.
