# Supplementary material for: Antepartum Antibiotic Treatment Increases Offspring Susceptibility to Experimental Colitis: A Role of the Gut Microbiota
Source: PLoS One. 2015 Nov 25;10(11):e0142536. doi: 10.1371/journal.pone.0142536 (PMC4659638; doi:10.1371/journal.pone.0142536)
Supplement: S3 Appendix — (DOCX) [file pone.0142536.s003.docx]

**Supporting information**

**S3 Text. Partial least square discriminant analysis and depth for alpha-diversity analysis**

Partial least square discriminant analysis (PLS-DA) is a particular case of partial least square regression analysis in which Y is a set of variables describing the categories of a categorical variable on X. In this case, X variables were bacterial taxa and Y was observations of different treatments compared together. To avoid over parameterization of the model, variable influence on projection value (VIP) was estimated for each genus, and genera with VIP < 0.5 were removed from the final model. R^2^ estimates then were used to evaluate the goodness of fit and Q^2^ estimate was used to evaluate the predictive value of the model.

For the alpha-diversity analysis, an even depth of approximately 27,000 sequences per sample for fecal samples collected before induction of colitis (ATB and Control groups), and 14,000 and 16,000 sequences per sample for colon mucosa and fecal samples of colitic mice (ATB-DSS and Control-DSS), respectively, was used for the calculation of diversity indices.
