# Supplementary material for: Antepartum Antibiotic Treatment Increases Offspring Susceptibility to Experimental Colitis: A Role of the Gut Microbiota
Source: PLoS One. 2015 Nov 25;10(11):e0142536. doi: 10.1371/journal.pone.0142536 (PMC4659638; doi:10.1371/journal.pone.0142536)
Supplement: S4 Appendix — (DOCX) [file pone.0142536.s004.docx]

**Supporting information**

**S4 Text.** **Prediction of functional metagenome**

The open source software PICRUSt (Phylogenetic Investigation of Communities by Reconstruction of Unobserved States), a computational approach for prediction of functional metagenome of bacterial communities using marker gene data and a database of reference genomes, was used on the Greengene picked OTUs to generate metagenomic data, and derive relative Kyoto Encyclopedia of Genes and Genomes (KEGG) Pathway abundance. KEGG data was analyzed using open source software STAMP (STatistical Analysis of Metagenomic Profiles).
