# Supplementary material for: Antepartum Antibiotic Treatment Increases Offspring Susceptibility to Experimental Colitis: A Role of the Gut Microbiota
Source: PLoS One. 2015 Nov 25;10(11):e0142536. doi: 10.1371/journal.pone.0142536 (PMC4659638; doi:10.1371/journal.pone.0142536)
Supplement: S1 Fig — In the ATB group, DSS caused a significant increase in diarreah (A), Blood in the feces (B) especially on day 2, 4, and 5, but did not significantly influence weight loss (C). (DOCX) [file pone.0142536.s005.docx]

**Supporting information**

**S1 Fig. Impacts of dextran sulfate sodium (DSS) and antepartum antibiotics on colitis induction and disease severity**. In the ATB group, DSS caused a significant increase in diarrhea **(A),** Blood in the feces **(B)** especially on day 2, 4, and 5, but did not significantly influence weight loss **(C).** Stool consistency, rectal bleeding and weight loss were analyzed by applying two-way ANOVA followed by Sidak multiple comparison post hoc.

**
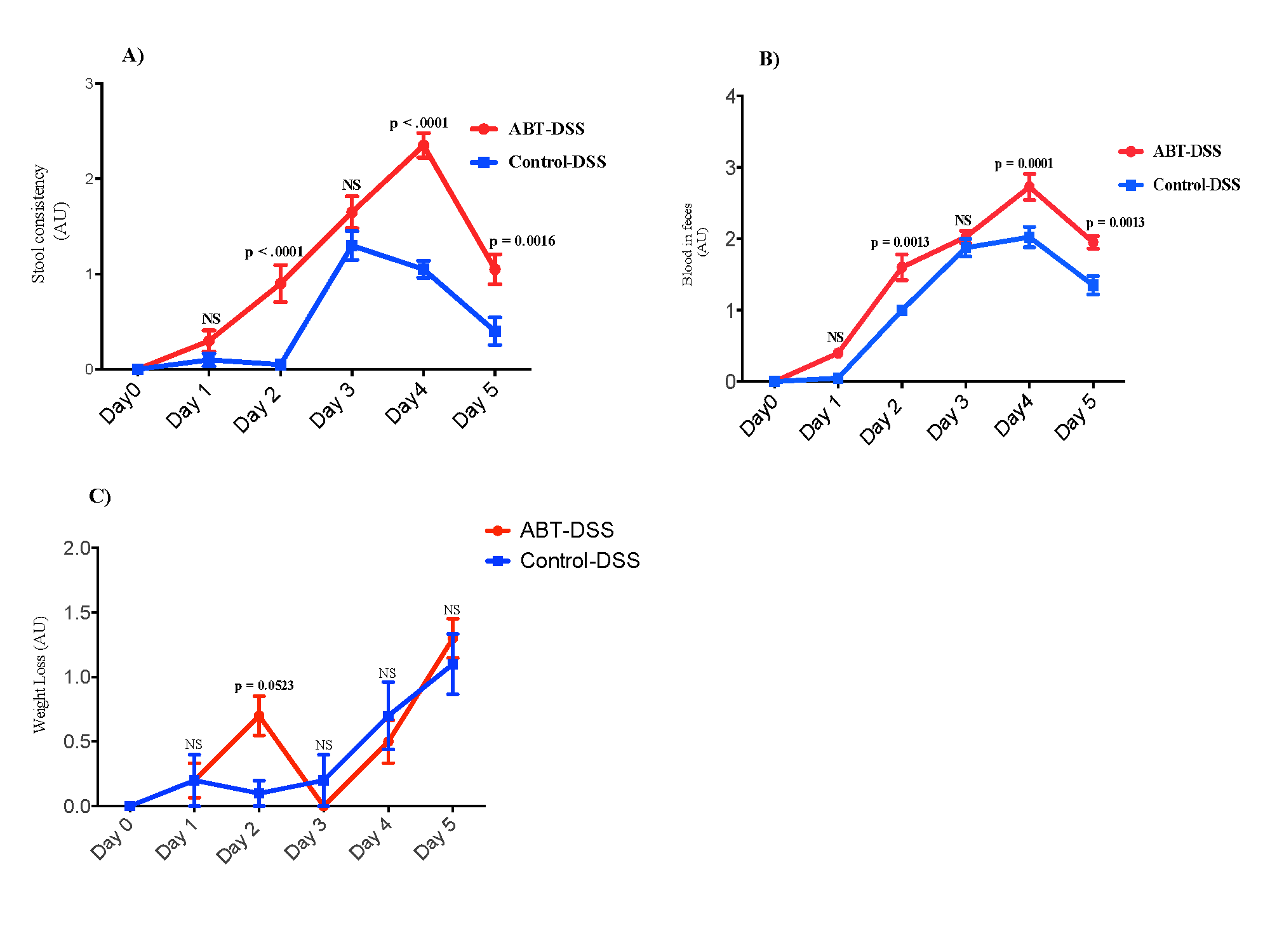
**
