# Supplementary material for: Antepartum Antibiotic Treatment Increases Offspring Susceptibility to Experimental Colitis: A Role of the Gut Microbiota
Source: PLoS One. 2015 Nov 25;10(11):e0142536. doi: 10.1371/journal.pone.0142536 (PMC4659638; doi:10.1371/journal.pone.0142536)
Supplement: S1 Table — Some taxa were only classified at the Phylum (p.), Class (c.), Order (o.), Family (f.), or Genus (g.) levels. Mean values only, no statistical analysis (DOCX) [file pone.0142536.s006.docx]

**Supporting information**

**S1 Table. Mean relative abundances of taxa in fecal samples collected just before induction of colitis.** Some taxa were only classified at the Phylum (p.), Class (c.), Order (o.), Family (f.), or Genus (g.) levels. Mean values, no statistical analysis.

| **Taxa** |  | **Mean relative abundance^*^** | | |
| --- | --- | --- | --- | --- |
|  |  | **Control** | | **ATB** |
| **--------------------------------------------Greater than or equal to 0.01%--------------------------------------------** | | | | |
| g. Adlercreutzia |  | | 0.109 | 0.115 |
| o. Bacteroidales |  | | 5.494 | 2.571 |
| g. Bacteroides |  | | 1.342 | 0.171 |
| g. Bacteroides acidifaciens |  | | 0.569 | 2.852 |
| g. Parabacteroides |  | | 0.094 | 0.203 |
| g. Parabacteroides distasonis |  | | 0.165 | 0.218 |
| f. Prevotellaceae |  | | 0.152 | 0.149 |
| g. Prevotella |  | | 3.574 | 8.411 |
| f. Rikenellaceae |  | | 3.219 | 1.328 |
| f. S24-7 |  | | 19.864 | 31.924 |
| g. Odoribacter |  | | 1.630 | 0.425 |
| o. YS2 |  | | 0.377 | 0.462 |
| g. Mucispirillum schaedleri |  | | 1.426 | 0.170 |
| g. Enterococcus |  | | 0.047 | 0.014 |
| g. Lactobacillus |  | | 24.655 | 18.634 |
| g. Lactobacillus reuteri |  | | 0.967 | 0.653 |
| o. Clostridiales |  | | 14.538 | 7.442 |
| f. Clostridiaceae |  | | 0.208 | 1.954 |
| g. Candidatus arthromitus |  | | 0.776 | 0.383 |
| g. Clostridium perfringens |  | | 0.001 | 0.034 |
| g. Dehalobacterium |  | | 0.397 | 0.165 |
| f. Lachnospiraceae |  | | 9.110 | 5.138 |
| g. Coprococcus |  | | 0.215 | 5.214 |
| g. Dorea |  | | 0.532 | 0.899 |
| f. Peptococcaceae |  | | 0.024 | 0.006 |
| g. rc4-4 |  | | 0.000 | 0.203 |
| f. Ruminococcaceae |  | | 1.337 | 0.889 |
| g. Oscillospira |  | | 1.892 | 1.025 |
| g. Ruminococcus |  | | 0.804 | 0.614 |
| f. Erysipelotrichaceae |  | | 0.038 | 0.052 |
| g. Allobaculum |  | | 0.019 | 1.305 |
| o. RF32 |  | | 0.101 | 0.610 |
| g. Sutterella |  | | 0.024 | 0.701 |
| g. Bilophila |  | | 0.057 | 0.060 |
| g. Desulfovibrio |  | | 0.936 | 0.000 |
| f. Helicobacteraceae |  | | 0.059 | 0.007 |
| g. Helicobacter |  | | 1.399 | 0.214 |
| f. Enterobacteriaceae |  | | 0.116 | 0.006 |
| f. F16 |  | | 0.534 | 0.460 |
| g. Anaeroplasma |  | | 0.644 | 1.290 |
| Unclassified |  | | 0.025 | 0.030 |
| **------------------------------------------------------Less than 0.01%-----------------------------------------------------** | | | | |
| g. Candidatus Microthrix parvicella |  | 0.0004 | | 0.0001 |
| g. Gordonia |  | 0.0002 | | 0.0002 |
| f. Micrococcaceae |  | 0.0005 | | 0.0002 |
| g. Arthrobacter |  | 0.0005 | | 0.0000 |
| g. Mycobacterium |  | 0.0000 | | 0.0003 |
| f. Coriobacteriaceae |  | 0.0019 | | 0.0017 |
| g. Bacteroides ovatus |  | 0.0010 | | 0.0084 |
| g. Parabacteroides gordonii |  | 0.0005 | | 0.0000 |
| g. Butyricimonas |  | 0.0002 | | 0.0001 |
| g. CF231 |  | 0.0000 | | 0.0006 |
| f. Flavobacteriaceae |  | 0.0013 | | 0.0000 |
| g. Chryseobacterium |  | 0.0003 | | 0.0003 |
| f. Sphingobacteriaceae |  | 0.0003 | | 0.0001 |
| p. Bacteroidetes |  | 0.0003 | | 0.0002 |
| f. Chitinophagaceae |  | 0.0002 | | 0.0001 |
| f. Saprospiraceae |  | 0.0012 | | 0.0003 |
| o. Streptophyta |  | 0.0102 | | 0.0048 |
| o. Bacillales |  | 0.0005 | | 0.0002 |
| g. Sporosarcina |  | 0.0006 | | 0.0002 |
| g. Staphylococcus aureus |  | 0.0005 | | 0.0000 |
| g. Staphylococcus sciuri |  | 0.0000 | | 0.0019 |
| o. Lactobacillales |  | 0.0005 | | 0.0006 |
| g. Aerococcus |  | 0.0003 | | 0.0002 |
| g. Facklamia |  | 0.0004 | | 0.0000 |
| f. Carnobacteriaceae |  | 0.0016 | | 0.0015 |
| g. Lactobacillus ruminis |  | 0.0005 | | 0.0002 |
| g. Streptococcus |  | 0.0070 | | 0.0001 |
| g. Streptococcus luteciae |  | 0.0033 | | 0.0000 |
| g. Turicibacter |  | 0.0005 | | 0.0023 |
| f. Christensenellaceae |  | 0.0017 | | 0.0013 |
| g. 02d06 |  | 0.0010 | | 0.0080 |
| g. Clostridium |  | 0.0018 | | 0.0096 |
| g. SMB53 |  | 0.0003 | | 0.0003 |
| g. Anaerofustis |  | 0.0019 | | 0.0019 |
| g. Blautia |  | 0.0004 | | 0.0002 |
| g. Epulopiscium |  | 0.0006 | | 0.0000 |
| g. Roseburia |  | 0.0002 | | 0.0002 |
| f. Veillonellaceae |  | 0.0000 | | 0.0004 |
| g. Phascolarctobacterium |  | 0.0002 | | 0.0001 |
| g. Coprobacillus |  | 0.0103 | | 0.0089 |
| c. Alphaproteobacteria |  | 0.0019 | | 0.0000 |
| f. Caulobacteraceae |  | 0.0003 | | 0.0007 |
| g. Rhodobacter |  | 0.0002 | | 0.0001 |
| g. Novosphingobium |  | 0.0002 | | 0.0001 |
| o. Burkholderiales |  | 0.0000 | | 0.0016 |
| f. Comamonadaceae |  | 0.0002 | | 0.0001 |
| g. Comamonas |  | 0.0005 | | 0.0005 |
| g. Thiobacterales |  | 0.0005 | | 0.0000 |
| f. Desulfovibrionaceae |  | 0.0063 | | 0.0000 |
| o. Myxococcales |  | 0.0000 | | 0.0002 |
| g. Flexispira |  | 0.0005 | | 0.0000 |
| g. Cellvibrio |  | 0.0005 | | 0.0010 |
| g. Aggregatibacter pneumotropica |  | 0.0010 | | 0.0000 |
| g. Acinetobacter |  | 0.0005 | | 0.0011 |
| g. Acinetobacter johnsonii |  | 0.0014 | | 0.0018 |
| g. Enhydrobacter |  | 0.0005 | | 0.0008 |
| f. Pseudomonadaceae |  | 0.0005 | | 0.0000 |
| g. Pseudomonas |  | 0.0006 | | 0.0017 |
| f. Xanthomonadaceae |  | 0.0002 | | 0.0003 |
| g. Stenotrophomonas |  | 0.0000 | | 0.0005 |
| o. RF39 |  | 0.0000 | | 0.0005 |
| g. Akkermansia muciniphila |  | 0.0000 | | 0.0062 |
| Unclassified |  | 0.0011 | | 0.0009 |

^*^ Mean values only, no statistical analysis
