# Supplementary material for: Antepartum Antibiotic Treatment Increases Offspring Susceptibility to Experimental Colitis: A Role of the Gut Microbiota
Source: PLoS One. 2015 Nov 25;10(11):e0142536. doi: 10.1371/journal.pone.0142536 (PMC4659638; doi:10.1371/journal.pone.0142536)
Supplement: S2 Table — Some taxa were only classified at the Phylum (p.), Class (c.), Order (o.), Family (f.), or Genus (g.) levels. Mean values only, no statistical analysis (DOCX) [file pone.0142536.s007.docx]

**Supporting information**

**S2 Table. Mean relative abundance of taxa in colon samples collected after induction of colitis.** Some taxa were only classified at the Phylum (p.), Class (c.), Order (o.), Family (f.), or Genus (g.) levels. Mean values only, no statistical analysis.

| **Taxa** |  | **Mean relative abundance^*^** | |
| --- | --- | --- | --- |
|  |  | **Control-DSS** | **ATB-DSS** |
| **--------------------------------------------Greater than or equal to 0.01%---------------------------------------------** | | | |
| p. Bacteroidetes |  | 0.026 | 0.022 |
| o. Bacteroidales |  | 7.558 | 2.132 |
| f. Bacteroidaceae |  | 0.012 | 0.031 |
| g. Bacteroides |  | 2.128 | 0.594 |
| g. Bacteroides acidifaciens |  | 0.060 | 1.037 |
| g. Parabacteroides |  | 0.079 | 0.517 |
| g. Parabacteroides distasonis |  | 0.355 | 0.873 |
| g. Parabacteroides gordonii |  | 0.002 | 0.021 |
| f. Prevotellaceae |  | 0.018 | 0.033 |
| g. Prevotella |  | 2.423 | 5.959 |
| f. Rikenellaceae |  | 1.463 | 1.096 |
| f. S24-7 |  | 8.256 | 14.656 |
| g. Odoribacter |  | 0.438 | 0.170 |
| o. YS2 |  | 0.305 | 0.090 |
| g. Mucispirillum schaedleri |  | 4.984 | 3.328 |
| p. Firmicutes |  | 0.101 | 0.140 |
| c. Bacilli |  | 0.050 | 0.017 |
| g. Enterococcus |  | 0.154 | 0.011 |
| f. Lactobacillaceae |  | 5.130 | 1.288 |
| g. Lactobacillus |  | 0.146 | 0.075 |
| g. Lactobacillus reuteri |  | 0.546 | 0.238 |
| g. Turicibacter |  | 1.476 | 1.578 |
| c. Clostridia |  | 0.639 | 0.643 |
| o. Clostridiales |  | 1.475 | 2.246 |
| f. Clostridiaceae |  | 0.099 | 0.116 |
| g. Clostridium |  | 6.510 | 16.759 |
| g. Clostridium perfringens |  | 0.069 | 0.284 |
| g. Dehalobacterium |  | 0.536 | 0.080 |
| f. Lachnospiraceae |  | 16.443 | 8.960 |
| f. Dorea |  | 0.054 | 0.016 |
| g. Gnavus |  | 2.600 | 0.339 |
| f. Peptococcaceae |  | 0.025 | 0.011 |
| g. rc4-4 |  | 0.000 | 0.053 |
| f. Peptostreptococcaceae |  | 3.221 | 0.008 |
| f. Ruminococcaceae |  | 0.802 | 0.627 |
| g. Anaerotruncus |  | 0.072 | 0.002 |
| g. Oscillospira |  | 1.686 | 0.750 |
| g. Ruminococcus |  | 0.475 | 0.494 |
| g. Ruminococcus flavefaciens |  | 0.000 | 0.257 |
| g. Adlercreutzia |  | 0.130 | 0.096 |
| f. Erysipelotrichaceae |  | 0.009 | 0.032 |
| g. Allobaculum |  | 0.178 | 0.594 |
| o. Erysipelotrichales |  | 0.089 | 0.022 |
| g. Coprobacillus |  | 0.021 | 0.005 |
| p. Proteobacteria |  | 0.070 | 0.119 |
| c. Alphaproteobacteria |  | 0.023 | 0.011 |
| o. RF32 |  | 1.157 | 0.884 |
| c. Betaproteobacteria |  | 0.012 | 0.015 |
| g. Sutterella |  | 0.367 | 0.416 |
| g. Bilophila |  | 0.085 | 0.133 |
| g. Desulfovibrio |  | 0.072 | 0.000 |
| g. Desulfovibrio C21_c20 |  | 0.857 | 0.000 |
| g. Helicobacter |  | 23.729 | 28.624 |
| H. hepaticus |  | 0.071 | 0.032 |
| f. Enterobacteriaceae |  | 0.015 | 0.025 |
| g. Escherichia |  | 0.911 | 0.937 |
| g. Aggregatibacter pneumotropica |  | 0.006 | 0.015 |
| f. F16 |  | 0.105 | 0.147 |
| g. Anaeroplasma |  | 0.519 | 1.178 |
| Unclassified |  | 1.097 | 1.021 |
| **-----------------------------------------------Less than 0.01%-------------------------------------------------------------** | | | |
| f. Geodermatophilaceae |  | 0.0018 | 0.0000 |
| g. Mycobacterium |  | 0.0000 | 0.0008 |
| f. Porphyromonadaceae |  | 0.0000 | 0.0002 |
| p. Cyanobacteria |  | 0.0014 | 0.0019 |
| f. Bacillaceae |  | 0.0005 | 0.0009 |
| f. Planococcaceae |  | 0.0014 | 0.0000 |
| o. Lactobacillales |  | 0.0146 | 0.0019 |
| g. Trichococcus |  | 0.0009 | 0.0000 |
| f. Enterococcaceae |  | 0.0027 | 0.0008 |
| g. Streptococcus |  | 0.0053 | 0.0000 |
| g. Clostridium butyricum |  | 0.0012 | 0.0003 |
| g. Oscillospira guilliermondii |  | 0.0005 | 0.0030 |
| f. Veillonellaceae |  | 0.0000 | 0.0006 |
| g. Mitsuokella multacida |  | 0.0000 | 0.0006 |
| f. Coriobacteriaceae |  | 0.0097 | 0.0051 |
| f. Caulobacteraceae |  | 0.0036 | 0.0015 |
| g. Phenylobacterium |  | 0.0004 | 0.0000 |
| o. Burkholderiales |  | 0.0014 | 0.0005 |
| g. Delftia |  | 0.0003 | 0.0011 |
| f. Desulfovibrionaceae |  | 0.0027 | 0.0000 |
| g. Desulfovibrio |  | 0.0004 | 0.0000 |
| f. Helicobacteraceae |  | 0.0009 | 0.0007 |
| c. Gammaproteobacteria |  | 0.0008 | 0.0007 |
| g. Acinetobacter guillouiae |  | 0.0000 | 0.0008 |
| f. Pseudomonadaceae |  | 0.0005 | 0.0000 |
| g. Pseudomonas |  | 0.0007 | 0.0017 |
| g. Stenotrophomonas |  | 0.0016 | 0.0000 |
| o. CW040 |  | 0.0000 | 0.0003 |
| c. Mollicutes |  | 0.0021 | 0.0080 |
| o. RF39 |  | 0.0000 | 0.0006 |
| g. Meiothermus |  | 0.0009 | 0.0000 |
| g. Thermus |  | 0.0003 | 0.0006 |
| g. Akkermansia muciniphila |  | 0.0011 | 0.0014 |

^*^ Mean values only, no statistical analysis
