# Supplementary material for: Antepartum Antibiotic Treatment Increases Offspring Susceptibility to Experimental Colitis: A Role of the Gut Microbiota
Source: PLoS One. 2015 Nov 25;10(11):e0142536. doi: 10.1371/journal.pone.0142536 (PMC4659638; doi:10.1371/journal.pone.0142536)
Supplement: S3 Table — Some taxa were only classified at the Phylum (p.), Class (c.), Order (o.), Family (f.), or Genus (g.) levels. Mean values only, no statistical analysis. (DOCX) [file pone.0142536.s008.docx]

**Supporting information**

**S3 Table. Mean relative abundance of taxa in fecal samples collected after induction of colitis.** Some taxa were only classified at the Phylum (p.), Class (c.), Order (o.), Family (f.), or Genus (g.) levels. Mean values only, no statistical analysis.

| **Taxa** |  | **Mean relative abundance*** | |
| --- | --- | --- | --- |
|  |  | **Control-DSS** | **ATB-DSS** |
| **--------------------------------------------Greater than or equal to 0.01%---------------------------------------------** | | | |
| p. Bacteroidetes |  | 0.095 | 0.041 |
| o. Bacteroidales |  | 12.051 | 2.822 |
| f. Bacteroidaceae |  | 0.064 | 0.012 |
| g. Bacteroides |  | 1.907 | 0.384 |
| g. Bacteroides acidifaciens |  | 0.103 | 1.470 |
| g. Parabacteroides |  | 0.075 | 0.845 |
| g. Parabacteroides distasonis |  | 0.317 | 1.361 |
| g. Parabacteroides gordonii |  | 0.005 | 0.027 |
| f. Prevotellaceae |  | 0.064 | 0.056 |
| g. Prevotella |  | 3.594 | 7.278 |
| f. Rikenellaceae |  | 1.537 | 2.222 |
| f. S24-7 |  | 9.755 | 13.587 |
| g. Odoribacter |  | 0.345 | 0.068 |
| p. Cyanobacteria |  | 0.094 | 0.052 |
| o. YS2 |  | 7.427 | 3.742 |
| g. Mucispirillum schaedleri |  | 1.509 | 0.402 |
| p. Firmicutes |  | 0.536 | 1.109 |
| c. Bacilli |  | 0.248 | 0.230 |
| o. Lactobacillales |  | 0.023 | 0.005 |
| g. Enterococcus |  | 0.023 | 0.007 |
| f. Lactobacillaceae |  | 4.298 | 1.993 |
| g. Lactobacillus |  | 0.544 | 1.205 |
| g. Lactobacillus reuteri |  | 0.341 | 0.790 |
| g. Turicibacter |  | 1.586 | 5.058 |
| c. Clostridia |  | 0.848 | 0.502 |
| o. Clostridiales |  | 2.181 | 3.495 |
| f. Clostridiaceae |  | 0.247 | 0.276 |
| g. Clostridium |  | 18.895 | 29.706 |
| g. Clostridium perfringens |  | 0.293 | 0.803 |
| g. Dehalobacterium |  | 0.326 | 0.028 |
| f. Lachnospiraceae |  | 14.267 | 5.411 |
| g. Dorea |  | 0.034 | 0.003 |
| g. rc4-4 |  | 0.000 | 0.163 |
| f. Peptostreptococcaceae |  | 4.735 | 0.070 |
| f. Ruminococcaceae |  | 0.608 | 0.598 |
| g. Oscillospira |  | 0.986 | 0.215 |
| g. Ruminococcus |  | 0.553 | 0.707 |
| g. Ruminococcus flavefaciens |  | 0.144 | 0.541 |
| g. Adlercreutzia |  | 0.043 | 0.032 |
| o. Erysipelotrichales |  | 0.065 | 0.049 |
| f. Erysipelotrichaceae |  | 0.036 | 0.163 |
| g. Allobaculum |  | 0.208 | 0.875 |
| p. Proteobacteria |  | 0.111 | 0.099 |
| c. Alphaproteobacteria |  | 0.250 | 0.140 |
| o. RF32 |  | 3.195 | 5.737 |
| c. Betaproteobacteria |  | 0.056 | 0.162 |
| g. Sutterella |  | 0.215 | 0.869 |
| g. Bilophila |  | 0.042 | 0.012 |
| g. Desulfovibrio C21_c20 |  | 0.232 | 0.000 |
| **Taxa** |  | **Mean relative abundance*** | |
|  |  | **Control-DSS** | **ATB-DSS** |
| g. Helicobacter |  | 0.749 | 0.600 |
| f. Enterobacteriaceae |  | 0.029 | 0.093 |
| g. Escherichia |  | 0.335 | 1.232 |
| f. F16 |  | 0.150 | 0.064 |
| g. Anaeroplasma |  | 0.520 | 0.662 |
| Unclassified |  | 3.011 | 1.859 |
| **-----------------------------------------------Less than 0.01%------------------------------------------------------------** | | | |
| g. Kocuria |  | 0.0000 | 0.0008 |
| g. Bacteroides helcogenes |  | 0.0058 | 0.0000 |
| f. Porphyromonadaceae |  | 0.0010 | 0.0071 |
| o. Streptophyta |  | 0.0002 | 0.0005 |
| o. Bacillales |  | 0.0036 | 0.0016 |
| g. Bacillus |  | 0.0014 | 0.0016 |
| g. Brevibacillus |  | 0.0005 | 0.0005 |
| g. Jeotgalicoccus |  | 0.0000 | 0.0006 |
| . Trichococcus |  | 0.0002 | 0.0000 |
| f. Enterococcaceae |  | 0.0026 | 0.0000 |
| g. Lactobacillus ruminis |  | 0.0015 | 0.0016 |
| f. Streptococcaceae |  | 0.0034 | 0.0000 |
| g. Clostridium butyricum |  | 0.0013 | 0.0010 |
| g. Butyrivibrio |  | 0.0009 | 0.0010 |
| g. Coprococcus |  | 0.0037 | 0.0016 |
| f. Peptococcaceae |  | 0.0084 | 0.0000 |
| g. Anaerotruncus |  | 0.0146 | 0.0016 |
| g. Oscillospira guilliermondii |  | 0.0016 | 0.0008 |
| g. Ruminococcus callidus |  | 0.0007 | 0.0027 |
| f. Coriobacteriaceae |  | 0.0037 | 0.0038 |
| g. Coprobacillus |  | 0.0099 | 0.0039 |
| f. Caulobacteraceae |  | 0.0002 | 0.0005 |
| o. Burkholderiales |  | 0.0006 | 0.0016 |
| f. Comamonadaceae |  | 0.0000 | 0.0010 |
| g. Desulfovibrio |  | 0.0075 | 0.0000 |
| g. Helicobacter hepaticus |  | 0.0078 | 0.0044 |
| c. Gammaproteobacteria |  | 0.0018 | 0.0026 |
| f. Pasteurellaceae |  | 0.0012 | 0.0023 |
| g. Aggregatibacter pneumotropica |  | 0.0048 | 0.0161 |
| g. Mollicutes |  | 0.0043 | 0.0093 |
| g. Akkermansia muciniphila |  | 0.0006 | 0.0000 |
| o. WCHB1-15 |  | 0.0000 | 0.0009 |

^*^ Mean values only, no statistical analysis
